# Software JimenaE allows efficient dynamic simulations of Boolean networks, centrality and system state analysis

**DOI:** 10.1038/s41598-022-27098-7

**Published:** 2023-02-01

**Authors:** Martin Kaltdorf, Tim Breitenbach, Stefan Karl, Maximilian Fuchs, David Komla Kessie, Eric Psota, Martina Prelog, Edita Sarukhanyan, Regina Ebert, Franz Jakob, Gudrun Dandekar, Muhammad Naseem, Chunguang Liang, Thomas Dandekar

**Affiliations:** 1 Department of Bioinformatics, Biocenter, University of Würzburg, Am Hubland, 97074 Würzburg, Germany; 2 Institut Für Mathematik Und Informatik, University of Würzburg, Am Hubland, 97074 Würzburg, Germany; 3 Fraunhofer Institute for Toxicology and Experimental Medicine - ITEM, Nikolai-Fuchs-Straße 1, 30625 Hannover, Germany; 4 Department of Microbiology, Biocenter, University of Würzburg, Am Hubland, 97074 Würzburg, Germany; 5 Pediatric Rheumatology/Special Immunology, University Hospital Würzburg, Josef-Schneider-Str. 2, 97080 Würzburg, Germany; 6 Orthopedic Center for Musculoskeletal Research, University of Würzburg, Friedrich-Bergius Ring 15, 97076 Würzburg, Germany; 7 Chair of Tissue Engineering and Regenerative Medicine, University Hospital Würzburg, Röntgenring 11, 97070 Würzburg, Germany; 8 Translational Center Regenerative Therapies, Fraunhofer Institute for Silicate Research (ISC), Röntgenring 11, 97070 Würzburg, Germany; 9 Department of Life and Environmental Sciences, College of Natural and Health Sciences, Zayed University, Abu Dhabi, UAE

**Keywords:** Cellular signalling networks, Computer modelling

## Abstract

The signal modelling framework JimenaE simulates dynamically Boolean networks. In contrast to SQUAD, there is systematic and not just heuristic calculation of all system states. These specific features are not present in CellNetAnalyzer and BoolNet. JimenaE is an expert extension of Jimena, with new optimized code, network conversion into different formats, rapid convergence both for system state calculation as well as for all three network centralities. It allows higher accuracy in determining network states and allows to dissect networks and identification of network control type and amount for each protein with high accuracy. Biological examples demonstrate this: (i) High plasticity of mesenchymal stromal cells for differentiation into chondrocytes, osteoblasts and adipocytes and differentiation-specific network control focusses on wnt-, TGF-beta and PPAR-gamma signaling. JimenaE allows to study individual proteins, removal or adding interactions (or autocrine loops) and accurately quantifies effects as well as number of system states. (ii) Dynamical modelling of cell–cell interactions of plant *Arapidopsis thaliana* against *Pseudomonas syringae* DC3000: We analyze for the first time the pathogen perspective and its interaction with the host. We next provide a detailed analysis on how plant hormonal regulation stimulates specific proteins and who and which protein has which type and amount of network control including a detailed heatmap of the *A.thaliana* response distinguishing between two states of the immune response. (iii) In an immune response network of dendritic cells confronted with *Aspergillus fumigatus,* JimenaE calculates now accurately the specific values for centralities and protein-specific network control including chemokine and pattern recognition receptors.

## Introduction

Signaling and cellular responses are difficult to model but central to understand differentiation, immunity and cellular processes and their development over time in general. In particular, the system state, the dynamics of cellular interactions and network control routes are notoriously difficult to model, though decisive to understand and influence cellular fate in health and disease. Boolean models look at the logical connectivity of a network, which result in a discrete time and state space. Very detailed is a description of the dynamics by differential equations^1^. However, detailed kinetics are rarely known. To fill this gap, semiquantitative approaches were introduced and include SQUAD^2^, BoolNet^3^, COPASI^4^ and CellNetAnalyzer^5^. JimenaE allows to simulate dynamically Boolean networks as well as a continuous version of Boolean models based on the SQUAD model^6^. In contrast to SQUAD, there is systematic and not just heuristic calculation of all system states. These specific features are not present in CNA, COPASI and BoolNet, whereas all these tools have in common to take a network topology and allow to predict the output behavior of the network.

However, severe challenges are systematic, exhaustive searches for the network state, interpolation between Boolean states, and identification of how does the network exert control. For these tasks, our software Jimena_E (for expert, in the following just “Jimena”) offers comprehensive analysis of regulatory networks and dynamics including swift and enhanced interpolation. Jimena excels by (i) rapid and exhaustive identification of system states, (ii) dynamical modelling of cellular interactions and describing amount and (iii) type of network control exerted by signaling proteins and receptors. Besides new optimized code JimenaE provides a number of additional features like network conversion into different formats, rapid convergence both for system state calculation as well as for all three network centralities analyzed and delivered by JimenaE.

We demonstrate Jimena´s abilities regarding (i) mesenchymal stem cell plasticity of differentiation pathways leading to osteocytes, chondrocytes and adipocytes. The analysis by Jimena includes major pathways and involved key regulators. (ii) Dynamics of cell interaction are analyzed by Jimena looking at *Arapidopsis thaliana* confronted with the pathogen *Pseudomonas syringae* DC3000. The Jimena modelling is validated by gene expression and protein interactome data sets. (iii) Different types of network control are elucidated in dendritic immune cells sensing *A. fumigatus*. We previously looked at application examples of our framework (old^7^ and recent^8^), but here we examine in depth the methods (see detailed sections at paper end), the mathematics, the advantages and limitations of the latest version of our Jimena framework, the new version JimenaE.

## Results

### Technical novelty of Jimena

While a majority of GRN-analysis algorithms are based on BooleCube or HillCube models (implemented in e.g. Odefy^9^), which result in a computing time complexity of Ω(2^n^) the algorithm used by our framework Jimena shows a computing time complexity of O(E) by relying on an analysis using Boolean Trees in combination with Binary Decision Diagrams to construct a working model all subsequent calculations are based on. This cut-down on calculation complexity leads to a highly efficient and fast framework whose execution only requires a standard desktop computer.

The algorithm used is an improvement of the SQUAD-algorithm^2,10^ that was furthermore extended by various useful features:

(i) While SQUAD was only able to calculate steady states of networks with a restricted size of ca. 100 nodes due to the discrete first calculation step, Jimena is able to skip the first analytical step, directly starting with the numerical calculation of the continuous dynamical system states which allows to analyze networks of any size.
(ii) Furthermore, we added a feature to analyze every single node of a network regarding its individual impact on the overall behavior of the network. These features are the node centralities as presented in Karl & Dandekar^6^.

Compared to this previous paper and the last version of the software, we have the following mathematical novelties:

(i) Stability of the solver for the differential equations such that also numerical tricky systems of differential equations can be solved. For this purpose, Jimena allows now for an adaption of the step size, please see “Mathematical Appendix” of the Supplementary material where we show that phenomena with a small SQUAD based example.
(ii) The integrals used to calculate the centralities are defined over a high dimensional space in which initial values of the nodes in a network are chosen. To cope with that high dimensionality, we use a Monte Carlo integrator. On the one hand, we can solve these high dimensional integrals quite fast, however due to its stochastic nature, results vary. We have provided a procedure how to apply the framework to get to any accuracy of the centralities that are appropriate. For details see the “The numerical procedure to calculate the centralities with Jimena was as follows” in the Methods section and Supplementary material for the procedure’s application. Consequently, we come to a practical compromise between accuracy and computational time.

The biological application examples presented here stress how we can now better elucidate biological control and regulation in specific biological networks.

However, we give next a basic introduction into the centrality concept for network control (Karl & Dandekar^6^). We define the state of a network to which it converges after a certain time of simulation as the network state. Taking two networks and simulate each network with the same set of initial values, we can define by the sum of the squared difference of the values of the nodes of the corresponding network states a measure for difference.

The Value Control Centrality (VC) represents the controlling leverage of the node onto the network. More specific, the VC value of a node expresses the importance of the initial value of the node (value the node has when the simulation starts) with respect to where the network converges. A high VC value identifies nodes that have an important impact of steering the network to different steady states since its values affect the convergence to specific steady states.

Total Control Centrality (TC) measures the vulnerability of a network to a knockout of this specific node and the severity of the consequences to the networks’ general properties. In other words, the TC value of a node accounts for the difference if the network contains the node or not. If the node is important, the network with and without the corresponding node will show a huge different behavior, resulting in e.g. that for the corresponding identical initial values of the nodes the networks converge to totally different steady states. The convergence to different steady states depending on the existence of the investigated node will providing a big TC value for the corresponding node.

The Dynamical Control Centrality (DC) as the third option gives an indication of a networks central signaling cascade(s) and its importance for a proper signal transduction^6,11^. More detailed, the DC value measures how important the node is as a signal transducer for the state the network converges. For this purpose, the inputs into the corresponding node are cut off. At the beginning of the simulation, the node is initialized with the value such that the convergence of the new network with the cut offs is as close to the original network as possible. This process is repeated for all the initial values for the other nodes. A high DC value indicates that rather the value during simulation is important, which is influenced by the information that is input through the incoming edges, than the initial value, which is not able to steer the network to the state of the original network although optimally chosen.

Hence, the signal modelling framework JimenaE allows to simulate dynamically Boolean networks. However, in contrast to SQUAD, there is systematic and not just heuristic calculation of all system states. These specific features are not present in CNA and BoolNet whereas all these tools have in common to take a network topology and allow to predict the output behavior of the network. JimenaE is our new expert extension of Jimena, besides new optimized code we provide a number of additional features like network conversion into different formats, rapid convergence both for system state calculation as well as for all three network centralities analyzed and delivered by JimenaE.

Table 1 gives a systematic comparison of Jimena with different popular modelling software: Odefy^9^ GNA (Genetic network analyser)^12^, CNA (CellNetAnalyzer)^5^, CNApy^13^, COPASI^4^, SQUAD^2^, JimenaE (this paper) and YANA^14^ so that the specific advantages and specifics of each software for its area become clear as well as the unique capabilities mentioned above and only addressed by JimenaE.

**Table 1:** Comparison of Jimena with different network modelling software.

	Odefy	GNA	CNA	CNApy	COPASI	SQUAD	JimenaE	YANA
Language	Matlab/Octave	Java	MATLAB/Octave	Python GUI	C++/QT	Java	Java	Java
License	Open-source	Non-open source	Non-open source	open source	open source	Non-open source	Open source	Open source
Algorithm	Boolean, ODE-based and stochastic polynomial interpolation	Boolean, piece-wise linear differential equation model	Plugins inc. Odefy	inc. Odefy	ODE-based, stochastic kinetics	Boolean, ODE-based polynomial interpolation	Boolean, ODEs, both SQUAD and OdeFy methods	/
Metabolic network analysis algorithm	/	/	Elementary-modes and pathway analysis	Elementary-modes and pathway analysis	Elementary-modes and pathway analysis	/	/	Elementary modes, extreme pathway and pathway analysis
Sensitivity analyses	/	/	/	/	x	/	/	/
Optimization and parameter estimation	/	/	x	x	x	/	x	x
Computation of steady state	x	x	x	x	x	binary decision algorithm	x	x
Model reduction techniques	/	x	/	/	x	/	/	/
Model markup language	Multiple SBML compat.; GraphML	SBML Qual /GNAml	SBML compat. Level 3	SBML compat	COPASI; SBML; SEEDML compat	SBML compat	GraphML	METATOOL; SBML compat
Data driven modelling	/	/	inference of signaling network topology	/	x	/	inference of external stimuli	fitting experimental data
Network centrality	/	/	Similar to degree centrality and betweennescentrality	/	/	/	Total centrality; Value centrality; Dynamic centrality for Node and connection respectively	/

/: not available.

x: present.

In our new version JimenaE, Jimena provides the export of files that allow for an immediate application of techniques from optimization for optimal external stimuli calculation, please see^15,16^. A number of new scripts supports network analysis and calculations by JimenaE. A tutorial describes the features of JimenaE https://www.biozentrum.uni-wuerzburg.de/bioinfo/computing/jimenae) and supplement contains all the scripts.

Specific mathematical novelties described here are now (i) the stability of the solver for the differential equations such that also numerical tricky systems of differential equations can be solved for a complex biological network (see “Mathematical Appendix”). (ii) The numerical procedure to calculate the centralities with Jimena is improved, providing a practical compromise between accuracy and computational time.

### Application of Jimena

We demonstrate here the application of the available features to analyze reconstructed gene regulatory networks. Jimena looks at the existing stable system states and additionally calculates the control centrality values for a measure of controllability, vulnerability and cascading functions for various cell types covering three different organisms. With its generic routines for network biology and network analysis, a clear advantage of Jimena is its broad applicability for scientific network analysis in botany, immunology, cell interaction and cancer research.

Jimena´s strengths are (i) rapid identification of system states, (ii) dynamical modelling of cellular interactions and calculating the amount and (iii) the type of network control exerted by signaling proteins and receptors. The new JimenaE has mathematical improvements, allowing higher accuracy in determining network states and, in particular, allows to dissect networks and identification of network control type and amount for each protein with high accuracy.

These strong features are demonstrated by three biological application examples (details on the involved biology and analysis of specific proteins and pathways are given in Supplementary material).

**Figure 1:**
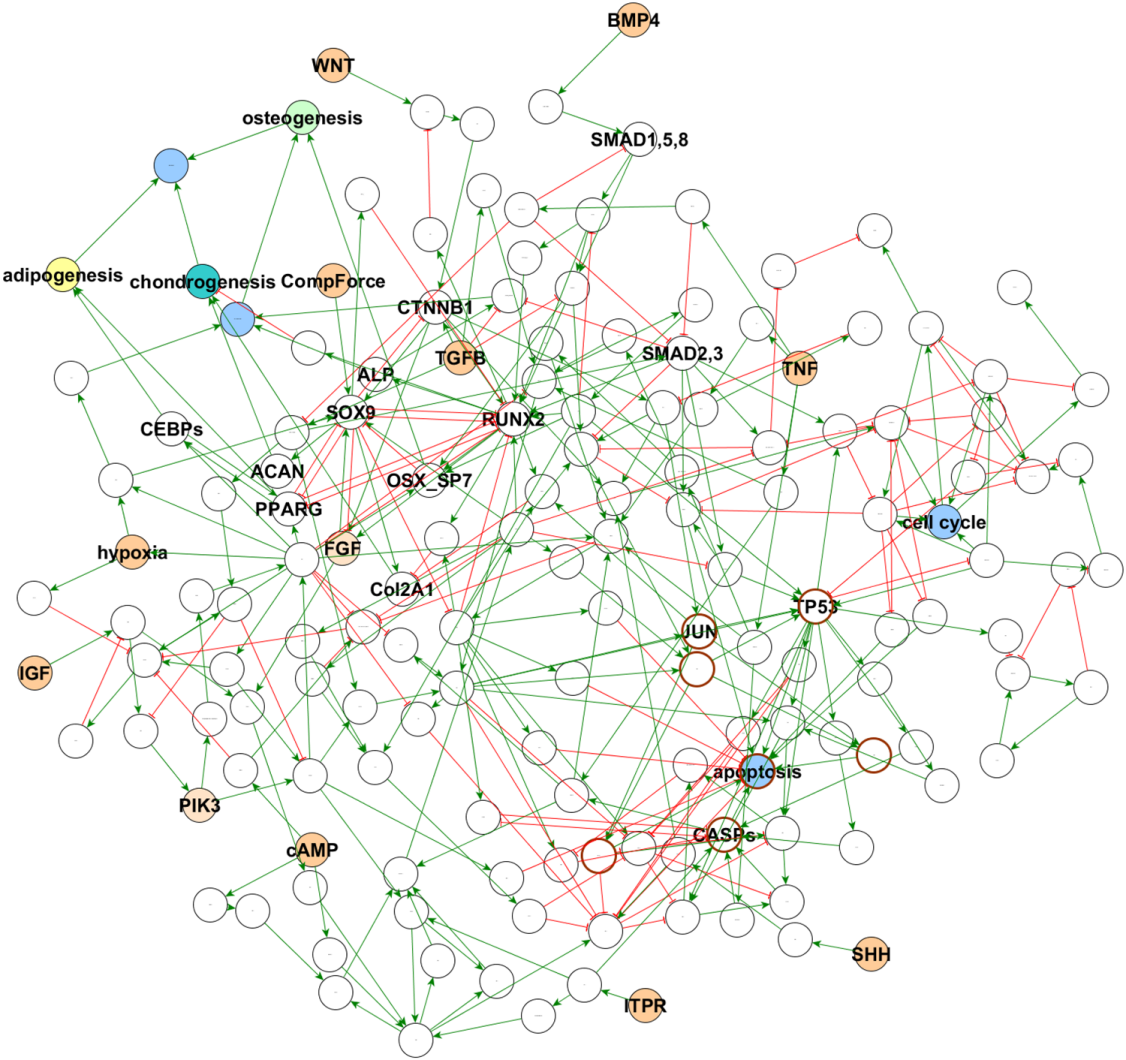
Regulatory network of mesenchymal stromal cell plasticity. Circles illustrate network nodes, characteristic interactions are colored (green arrows: activation; red lines with T-shaped endpoint: inhibition). Blue nodes highlight systemic responses of the modeled cell. All potential stimuli nodes are colored orange. The targeted cellular functions of interest adipogenesis (yellow node), chondrogenesis (cyan) and osteogenesis (green) are uniquely accentuated.

**Figure 2:**
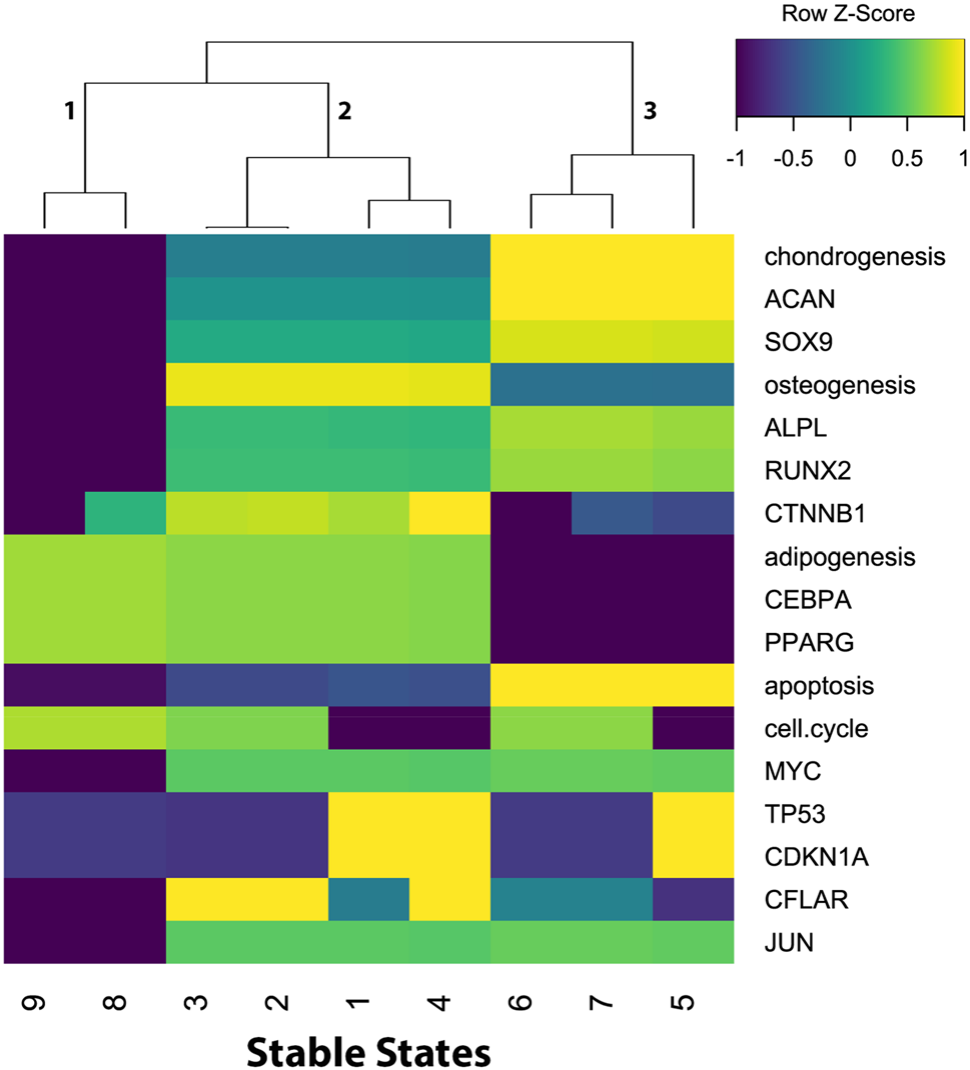
Revealing adipo-, chondro- and osteogenic differentiation pathways by Jimena. Heatmap illustrating all existing stable steady states in the MSC network. There are genes presented in a heatmap, but also pathways represented (see results). Jimena allows to represent also a whole pathway as a single read-out node in the network. This is a simplification but works for instance in this application example well. Each cell shows the log10 transformed color coded activity in comparison to the mean activation of each node. Blue illustrates downregulation of up to a logFC = −1 while yellow codes an upregulation of up to logFC =  + 1. Green codes for no significant or only slight changes in activity. The dendrogram sorts the stable steady states into three different groups each standing for a specific activity pattern: group 1 with inactive osteogenesis and chondrogenesis and neutral to slight upregulation of adipogenesis; group 2 presenting an upregulation of osteogenesis and group 3 with active chondrogenesis and some osteogenesis markers while adipogenesis is downregulated.

Cellular Differentiation pathways and networks are analyzed with Jimena looking at pluripotent human mesenchymal stem cells (MSC; also called mesenchymal stromal cells) into different cell types of osteocytes, chondrocytes and adipocytes. The regulatory network in MSCs is given in Fig. 1. Our new version JimenaE features compared to previous Jimena versions improved stability of the solver and more reliable modelling of the network and its centralities. Hence, also this complex model and the different cellular differentiation pathways can easily be calculated and modelled. This includes involved pathways, key regulators and transcription factors exploiting the accurate centrality values. As a particular striking new insight using JimenaE, the high plasticity of the multipotent stem cells are revealed (depends on accurate calculation of the different system states). We can thus see the high number of ground states accessible to the MSC and how the number of accessible ground states is reduced when differentiation happens into adipocytes, chondro- and osteoblasts (Fig. 2) with specific pathway activation patterns according to the experimental data (see supplement). The network and the dynamic simulations were never published before, however, previous published gene expression data^17^ validate our findings (see supplement for details). Moreover, Kerkhofs et al.^17^ presented in their work already a qualitative model of network, steady states and the role of autocrine loops for the growth plate network. The new input from JimenaE here is that the detailed network is modelled. We use our own network, but the software runs also on any other preferred network topology including the model in^17^.

**Figure 3:**
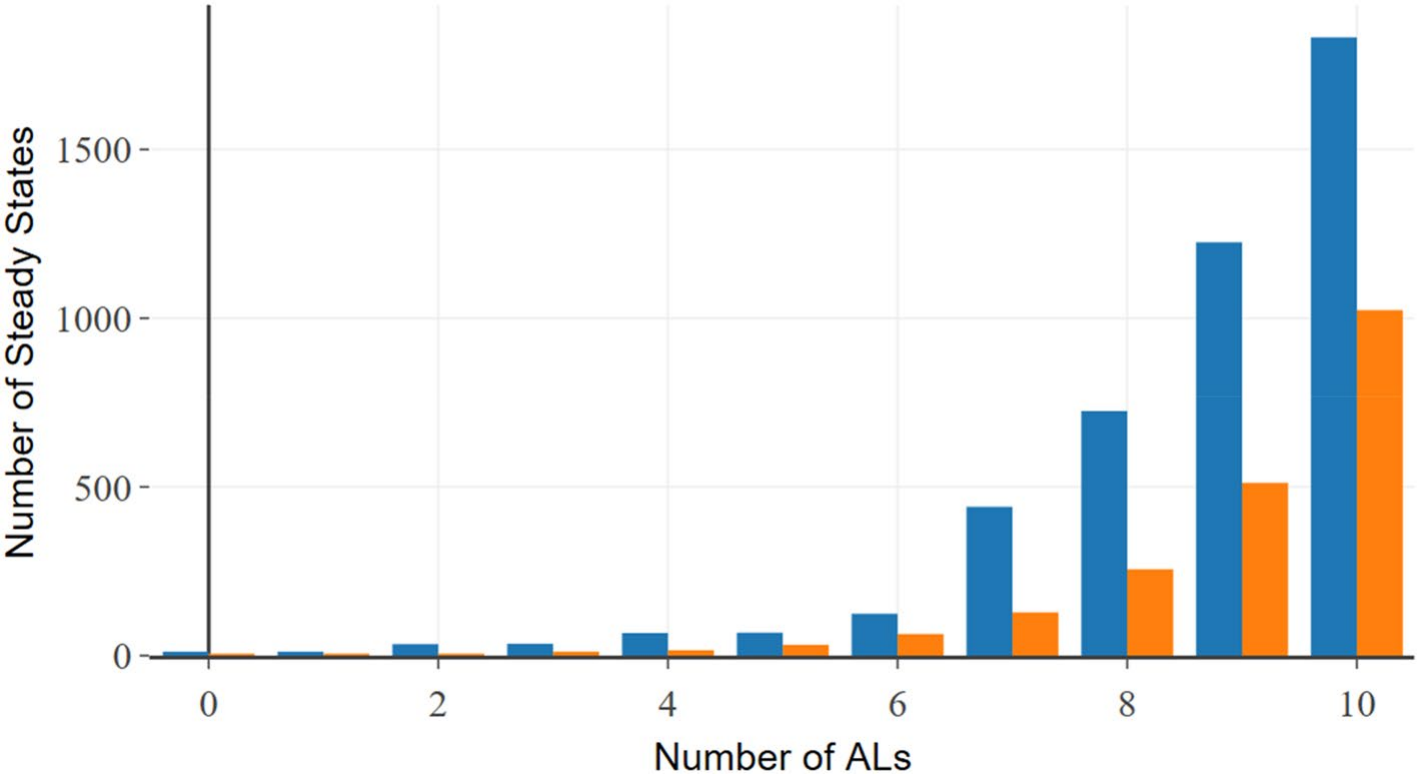
Autocrine loops and stromal cell plasticity enhancement by these. Plasticity of regulatory networks. The histogram illustrates the number of equilibria states of the MSC-network model in dependency of a variable number of autocrine activating stimuli. Orange: The base network illustrated in Fig. 1. implies the original mode without any autocrine activations. Blue: Subsequently known external stimuli were set as autocrine.

Figure 1 illustrates the regulatory network of mesenchymal stromal cell plasticity which is modelled here using Jimena. Next the mesenchymal stromal cell can exist in different system states shown here as a heat map (Fig. 2) to reveal the three different adipo-, chondro- and osteogenic differentiation pathways as identified by Jimena. The heat map demonstrates not only the stable cellular states, but we can monitor the specific activities of key regulator genes such as Sox9, RUNX2, MYC, p53 and Jun and how they change in one of the specific states, all this is now accurately calculated by JimenaE. Finally, JimenaE due to its mathematical improvements can also now analyze and describe the complex stromal cell plasticity including enhancement by autocrine loops which allow the MSC to access more system states (Fig. 3). JimenaE thus allows now to study specific effects of individual proteins, removal or adding interactions (or autocrine loops) and accurately quantifies resulting effects: The resulting type of network control for individual proteins and their network effect, as well as the effects on the number of system states, a nice addition to previous studies. Supplement gives detailed results on this network and its proteins.

**Figure 4:**
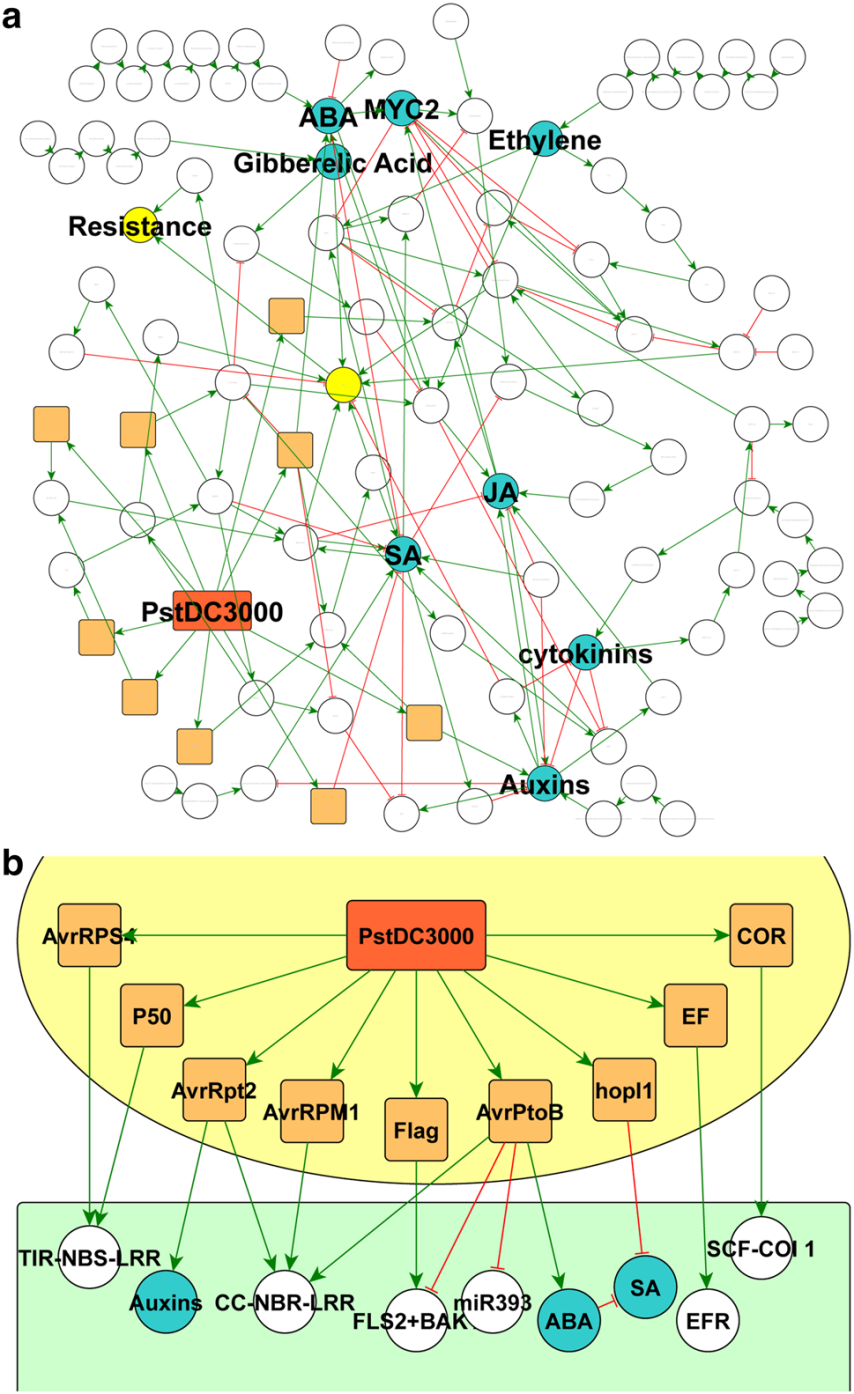
Network model of *A.thaliana* during infection and its key interactions with pathogen *Pst* DC3000. (**A**) Interaction model of plant immune system and its interactions with compounds of *Pseudomonas Syringae* DC3000 (*Pst*DC3000). Nodes with rounded rectangular framings colored orange illustrate factors secreted by PstDC3000 (red). Nodes with elliptical frames represent proteins of the host organism *A.thaliana*. Nodes colored blue display important plant hormones that possess an essential function in the immune response of the plant while network components of light green color stand for systemic responses of the plant. (**B**) *Pathogen perspective:* Using the yEd Editor we show here for the first time the pathogen perspective: *A. thaliana* is just another environment, specific interactions regulate Pst responses. Orange colored components illustrate parts of the PstDC3000s organism: orange-red: PstDC3000; orange: PstDC3000 proteins that directly interact with *A.thaliana* factors. White nodes show general *A. thaliana* proteins and complexes while blue nodes illustrate central plant hormones. Green arrows stand for activating influences; red lines with T-shaped ending depict inhibitory edges.

**Figure 5:**
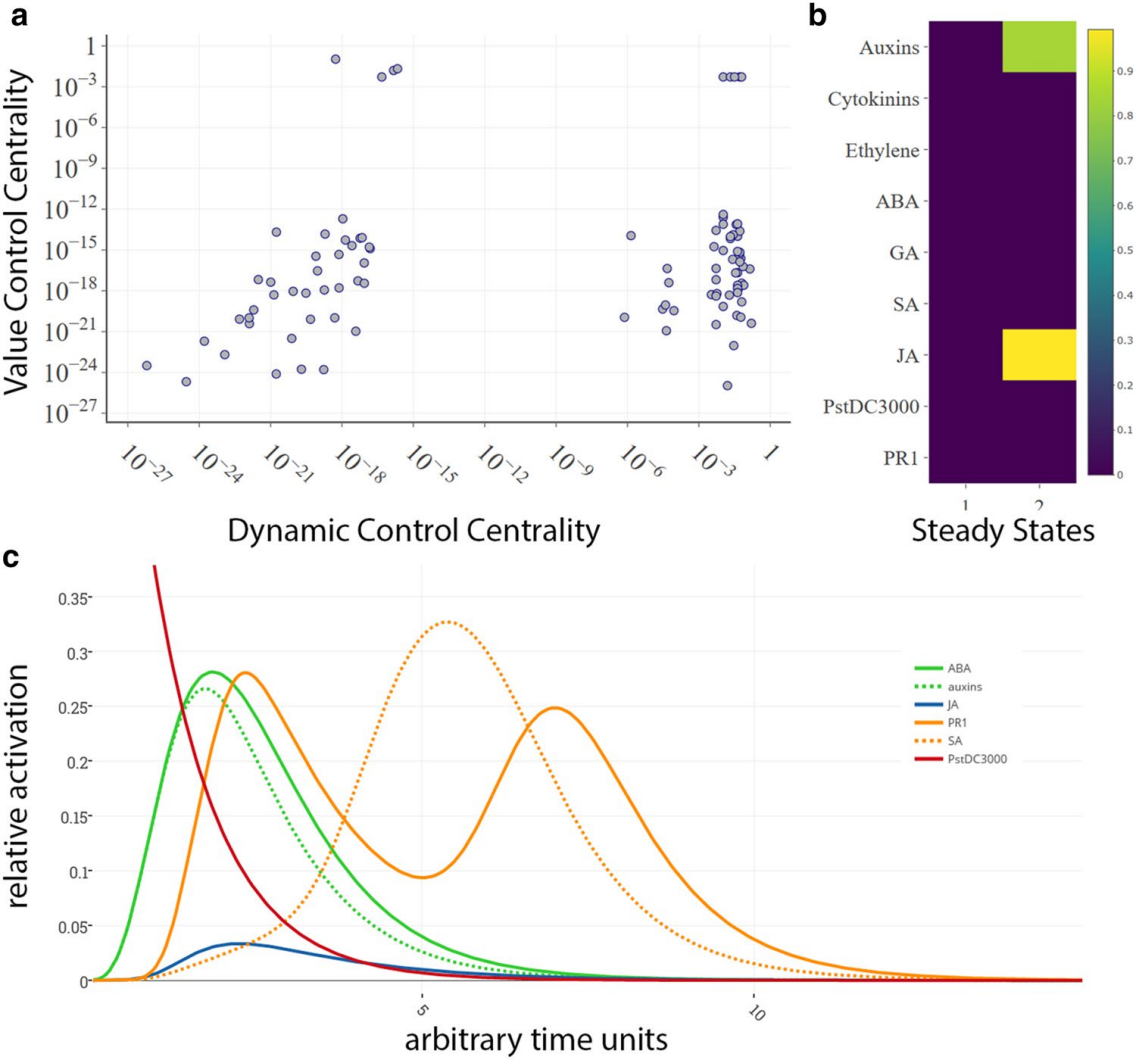
Control Centrality and Stable Steady States in *A. thaliana* quantifies jasmonic acid and cytokinine responses and modulation. (**A**) Value Control Centrality plotted against Dynamic Control Centrality showing nodes of high general controlling influence on the network and a strong involvement in core pathways of the network in the right upper corner. (**B**) Heatmap of relative activity values of the possible stable steady states in the *A. thaliana* immune response. The range of activity is illustrated by a color scale spanning from yellow (fully active node, 1.0) via green (intermediate activity, ~ 0.5) to dark blue (no activity, 0.0). (**C**) Dynamic simulation of the immune response of *A.thaliana* to initial full activation of *PstDC3000* (1.0, red line) illustrating the zig-zag-shaped activity of immune marker PR1 (orange line). The primary response to PstDC3000 is constituted by ABA (green line) and auxins (green discontinuous line) while the secondary peaking response of PR1 is preceded by a peak in salicylic acid activation (orange discontinuous line) (GEO Datasets used: GSE3984: hormonal stimuli of salicylic acid: cytokinin (GSE5520)).

The dynamics of cellular interactions are illustrated using Jimena by looking at *Arapidopsis thaliana* confronted with the pathogen *Pseudomonas syringae* DC3000. We published earlier a treatment of the dynamics of this infection by Jimena^18^ as well as simulation protocols for immune dynamics in plants using this network^8^. The network model used for the Jimena simulation is shown in Fig. 4A in the published view. What does bring JimenaE here new compared to previous? First, we improved the network editor and connected scripts for interchanging network formats. We demonstrate this with Fig. 4B. We show here for the first time the pathogen perspective and its interaction with the host. Next, a detailed analysis of the textbook zig-zag model of plant-pathogen interaction is possible, replacing the simple zigzag description of plant-pathogen interaction, pattern recognition triggered immunity, elicitor secretion by the pathogen and finally elicitor triggered immunity by the plant by detailed modelling of the involved protein networks using dynamical modelling of responses and protein activities using Jimena (see Fig. 5).

The mathematical refinements and software improvements of JimenaE allow now a detailed analysis on how plant hormonal regulation stimulates specific proteins and who and which protein has which type and amount of network control. This is summarized in Fig. 5A. Moreover, this leads to different system states of the *A. thaliana* plant cell. A detailed heatmap of the *A.thaliana* distinguishing between two states of the immune response is now possible applying JimenaE and shown in Fig. 5B. The resulting dynamic simulation of the immune response of *A.thaliana* to *PstDC3000* by Jimena is shown for illustration in Fig. 5C. As mentioned earlier in previous publications^8,18^ the simple zigzag model of immune response is replaced by dynamical network simulation of all involved nodes. However, with JimenaE now the specific values calculated, both regarding centralities and protein-specific network control are now more accurate. The primary response to PstDC3000 is now accurately quantified, considering hormone responses (e.g. ABA, auxins; Fig. 5B) and the secondary peaking response of PR1, preceded by a peak in salicylic acid activation. In the results we give only selected trajectories for proteins of interest, but Jimena calculates responses for all proteins of the network, and JimenaE is now more accurate and allows also to monitor exactly the three different types of network control for each protein. The resulting modelling results were further validated by gene expression and protein interactome data sets (see also supplement).

**Figure 6:**
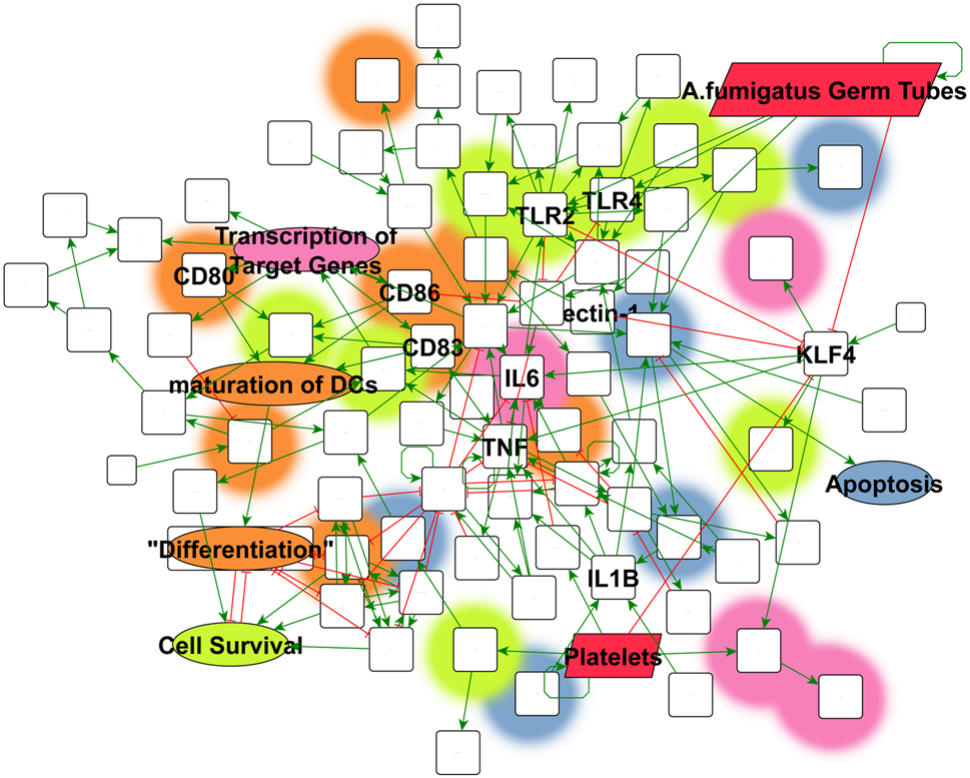
Network topology in human dendritic cells of key pathways in *A. fumigatus* response. Regulatory network model showing the signaling pathways central to dendritic cell immune answer in presence of *A. fumigatus* under influence of platelet rich plasma (PRP). The cellular function of proteins according to pantherDB is highlighted by different overlay colors: red highlights stimuli nodes of the network, light-red inflammatory proteins, blue illustrates proteins with regulatory influence on apoptosis, while orange marks proteins connected to developmental processes. All proteins directly connected to cellular immune response regulation are colored green. All interactions are covered by two different types: inhibitory (red lines with T-shaped endpoint) and activations (green arrows).

Different types of network control can be better revealed and networks correctly dissected applying JimenaE. This is exemplified by looking at dendritic immune cells sensing *A. fumigatus*. This network had also been previously analyzed by us using Jimena^7^ but we provided at that time only a first semi-quantitative simulation of the immune dynamics focusing on the involved immune receptors. Jimena allows for instance to model the stimulatory influence of platelet rich plasma (PRP) for the response to *A. fumigatus*^7^. Figure 6 shows the network topology of key pathways in human dendritic cells involved in the response to *A. fumigatus* as analyzed applying JimenaE. Here JimenaE allows now to dissect accurately the immune network and describe the network control, differentiating between dominating and less important nodes and identify their type(s) of network control. Biologically this implies besides pattern recognition receptors also chemokine receptors and connected pathways involved in the defense process. Details in supplement discuss the specific proteins and immune signaling involved and their contributions.

Supplement goes over all the examples in detail and confirms that all analysis features of JimenaE (Table 1, results) can be applied to the three biological example networks given and gives for each concrete results regarding pathways, dynamics, control centralities and validation data. All networks are given as XML/graphml files in supplement and the excel files give references for the involved nodes and their connectivity in the three studied networks. All three features (i)-(iii) can be found for each network, this is demonstrated in detail in the supplement.

## Discussion

Systems biology modelling of protein and molecular interaction networks is an important key to understand cells, molecules, and the resulting system effects^19,20^. Well known software includes SQUAD ^2^ and Cell Net Analyzer^5^ for Boolean Semiquantitative Modelling as well as related packages for dynamic network modelling such as COPASI^4^ and the systems biology R suite.

Jimena has own specific advantages by focusing on network centralities and how the network is steered and controlled, it offers different interpolation methods within the network and allows robust searching to identify the stable system states.

Although alternative software solutions also provide the possibility to calculate steady stable system states (like Odefy), Jimena shows superior performance due to its two-step algorithm consisting of discrete followed by continuous steady state analysis^11^. Compared to its unofficial precursor SQUAD, Jimena further extends the functionality by optional calculation without discrete stable states, allowing for the analysis of significantly bigger networks (> > 100 nodes).

Another included feature allows the readout and even the alteration of the complete differential equation built based on the network topology for the use in alternative mathematical solvers (e.g. MATLAB, Mathematica) to verify the Jimena inbuilt solver but even extend the capabilities to the calculation of optimal activation patterns by external stimuli with techniques from mathematical optimization in order to shift from one steady state to another^15^.

The knowledge-based network reconstruction includes all data curated in interaction databases and is iteratively verified by gene expression data resulting in a well characterized directed interaction model valid for functional analysis and subsequent simulations with the target of in silico prediction of biological systems. Alternative approaches using reverse engineering techniques via proteomics, genomics and interactomics that state a promising possibility of future network reconstruction for systems biological analysis, still struggle with the identification of directed interactions for regulatory simulations.

Jimena provides a reliable, easy to use network analysis approach for a better understanding of biological systems by semi-quantitative in silico prediction of their behavior due to external and internal perturbations and involved network centralities. Further applications of Jimena not demonstrated here include speed up of the development and prediction of reliability of new medical therapeutic strategies as well as agricultural pest control.

### Comparison to related modelling methods

We discuss here mathematical arguments of the three programs closest to Jimena in extension of the Table 1 feature comparison given in results:

We wanted to compare JIMENA to other software (eg. COPASI) that focus on kinetic ODE models. There are analogies and differences between such models vs. logic-based continuous models, in particular, the kinetic models include accurate parameters describing the kinetics. Our aim for the comparison is to compare a number of tools for network analysis, no matter whether they are with kinetic ODEs or logic-based continuous models. Those models we picked are just popular and with broad capabilities for network analysis, one has to be aware that this is no complete overview on all available tools.

SQUAD is a simulation tool for simulating ordinary differential equations with a specific model, please see e.g. Di Cara et al.^2^ Eq. (2).

It does not provide the dynamic centrality values for nodes. Furthermore, in contrast to Jimena, which is continuously extended, see e.g. the switch analyzer in JimenaE, SQUAD does not seem to be maintained any more: See the link they provide to download SQUAD but the domain does not exist any more: http://www.enfin.org/squad.

Boolnet is a tool that simulates networks where the nodes can only take two values^3^. However, in our case we are interested in building networks that account for continuous dynamics to study also the influence of intermediate values of nodes.

Furthermore, to the best of our knowledge Boolnet has no tools implemented to simulate centrality values that are similar to ours.

Cellnetanalyzer (http://www2.mpi-magdeburg.mpg.de/projects/cna/general_information.html) can be used for metabolic flow network construction and analysis. According to the manual (http://www2.mpi-magdeburg.mpg.de/projects/cna/manual_cellnetanalyzer.pdf), Cellnetanalyzer can be used to analyze multistate signal-flow networks, in particular in terms of how the network has to be designed to fulfil certain properties the user desires, like a desired behavior that the network is supposed to show. Consequently, the Cellnetanalyzer is a valuable tool to be used upstream of Jimena to reverse-engineer a network structure that has desired steady states. After the modelling, the importance of each node in the network can be analyzed with Jimena to get insights into their function for the network, e.g. if a node dominates the network rather by its value or by transmitting information/signals (VC,DC) or what node is the most important one for having the desired behavior (TC).

### Limitations and future extensions

One limitation of Jimena is the coupling of the network topology to measured data. The actual version uses the SQUAD model^10^ where the parameters can only be set for all nodes and not individually. Consequently, the network topology is manually adapted such that the values of the nodes in a steady state fit the experimental observations in a semi-quantitative manner.

Consequently, future work is to extend Jimena in that sense that different models, each based on the information of the network topology, can be interchanged where the parameters are fit with a “best parameter fit” method according to measured data. This data could be RNA counts or Western-blot data to associate an activity to each node for each experiment. Such a multi-experiment fitting software package, called D2D (Data to dynamics), see Raue et al.^21^, is provided e.g. under https://github.com/Data2Dynamics/d2d for Matlab or https://github.com/dkaschek/dMod for R via corresponding git repositories.

This mentioned limitation can be circumvented with a symbiotic fusion of D2D and Jimena where a model export from D2D and an import into Jimena can be used to further analyze the model after the model best fitting to the data has been systematically identified in D2D. The analysis can comprise steady states and network centrality analysis.

## Methods

Each biological process in a living organism can be broken down into regulatory interactions that shape their cellular as well as their systemic functions. In light of the increasing knowledge on functional and regulatory interactions between genes and their gene products like miRNAs and proteins, the necessity of their efficient and reliable analysis regarding their intrinsic impact and dependencies on any organism is getting urgent. In silico analysis and simulation of Gene Regulatory Networks (GRNs) repeatedly demonstrated their potential to be a central instrument to tackle this challenge, allowing for the prediction of a network in course of regulatory influences on any of its nodes and furthermore shortening the effort in experimental approaches in the investigation of every systemic issue. However, the transfer of in silico results onto experimental data still remains difficult. Furthermore, the complexity of regulatory network models demands an exponentially growing computing power, depending on the networks’ scale.

While the majority of GRN-analysis algorithms calculate a networks properties using BooleCube or HillCube models, resulting in a computing time complexity of Ω(2^n^), the algorithm used by Jimena analyzes the network using Boolean Trees in combination with a binary decision diagram to construct the working model, reducing the computing time and space to O(E), E number of edges of the network/graph. This cut-down of required computing power results in a framework capable of calculating a GRNs system states and the property of single nodes on any personal computer.

The algorithm used is in principle an improved method first introduced by Mendoza et al.^10^ and then implemented in the software SQUAD^2^. Additionally, Jimena includes features to indicate the network robustness and controllability using various control centrality values for every node and edge. This allows the user to estimate the biological relevance of the network.

Furthermore, Karl & Dandekar^6^ described a novel algorithm to calculate the centrality of nodes and edges inside a network model allowing for a measure of their controlling power on the total model and thus enabling us to identify major hub genes as well as genes which are prone to influence the whole network in case of their manipulation. We describe here the application of the analysis of total control centrality and value control centrality to get a factor of the nodes properties of vulnerability as well as controllability of both, all single nodes as well as the global network topology.

### The numerical procedure to calculate the centralities with Jimena was as follows

For the different type of centralities different integrals are to calculate^6^. In the corresponding Jimena implementation, the integrals are calculated with a Monte Carlo method where supporting points are randomly chosen. Between two supporting points the integrand’s value is taken as a constant. In order to approximate the integral values properly, a sufficient number of iterations (number of supporting points) is necessary in the Jimena Software package. The heuristic behind this procedure is that if we use a sufficient large number of supporting points, the numerical value only changes at digits that correspond to an accuracy that we define as sufficient. If we increase the number of iterations for the calculations of the node centralities in each sweep, we observe convergence of the values of the centralities for each node, meaning that the absolute value of the difference of the centralities of each node from one sweep to the next one decreases. For a sufficient large number of iterations, the absolute value of the difference between the centrality of each node of two different runs falls below any accuracy threshold. This means for a practicable use of the Jimena Software with respect to centrality calculations we increase the number of iterations and run the calculations twice. If the corresponding values are equal up to that digit that the calculation is supposed to be reliable, we have found a reasonable number of iterations that result in a sufficient accuracy of the values of the centralities of the nodes.

Those factors give us a reliable information on the quality and transferability of those networks onto biological systems in order to recommend experimental approaches based on the analysis’ results.

We demonstrate the validity of our approach using various different organisms from different kingdoms: (1) a differentiation model of human multipotent stromal cells (MSC) into chondrocytes, adipocytes and osteoblasts. System state analysis (23 states) shows the dependency of the differing cell fates on important known differentiation marker genes, but also the impact of growth factors on their determination. The inclusion of autoactivation of receptors and growth factors shows plasticity (> 10,000 states) and stability of the differentiated cell types. System and pathway changes are validated by gene expression data from differentiated chondrocytes. (2) A signaling pathway illustrating human dendritic cells in response to fungal infection (*Aspergillus fumigatus*). Jimena identifies the central control nodes in the network and delineates total control centrality, value control centrality as well as dynamic control centrality. (3) Jimena signaling models are efficiently imbedded in CAN simulations, this is shown for different T-cell pools (see TCell_CannedModel.png).

Given the knowledge on intercellular signal transduction the regulatory signal flow can be calculated using a “canned” model network where each single can consist of an intracellular signaling network that can communicate with adjacent cells via signaling molecules. This allows for the simulation of the regulatory mechanisms of simple cell populations.

The exemplary model consists of multiple different T-cells. Each of those T-cells is built up by a simple regulatory pathway that is known to control T-cell fate. The Boolean network model of the different regulatory pathways can now be modelled in its dynamics. This is supported by Jimena by the option to select various different simulation methods in order to compare the differences in their dynamic simulations and identify the optimal fitting paradigm.

### Reconstruction of a network model

The interaction information used for the reconstruction of the network topology can be collected by the aid of various different data sources. Curated databases respective protein–protein-interaction data are the preferable source in order to reconstruct the important connections and provide a quick and easy possibility to build an initial network model. The interaction data has to contain information on the characteristic of the connection in order to make use of it in the subsequent protocol. Hence it may be possible to convert the raw information acquired from a database into a Jimena conform data structure: The network can be only properly analyzed using ‘activating’ and/or ‘inhibiting’ interactions.

The databases suited the best for general network reconstruction purposes are (a) KEGG (Kyoto Encyclopedia of Genes and Genomes^22,23^), (b) STRING (Search Tool for the Retrieval of Interacting Genes/Proteins^24,25^) as well as (c) the REACTOME Pathway Database^26^. Depending on the respective organism other protein-protein-interaction (PPI) databases can cover additional data and hence lead to better interaction data and thus in a higher quality of the network model.

We confirmed the reconstructed network topologies, especially disputed interactions, using recent publications to ensure its validity and prevent mistakes and parallel signal flow during the analysis and simulation.

All interactions were compiled in a text file (“.txt”) containing a table of the format (columns should be separated by “\t” resp. “tabulator”-key):

**Table:** 

Input Node	Interaction	Output Node	Reference
Abc	activation	Xyz	Kaltdorf et al. (2015)
Xyz	inhibition	Abc	Kaltdorf et al. (2015)

In order to create the network file for analysis and simulation with ‘Jimena’, we created a perl script to compile the previously created text file containing all the interaction information into a “.graphml” file. Also some other tools to convert between standard formats in systems biology are provided by *The Systems Biology Graphical Notation* group a thorough list of available tools for conversion can be found here https://sbgn.github.io/software. The “.graphml” format is equivalent to a specialized xml format that can be interpreted and edited by the yEd graphing software.

Network edges and nodes including their coloring are automatically created by the perl-script. Once the .graphml file has been compiled its layout can be modified in yEd to create a structured network graph. The ‘Layout’ drop-down menu of yEd presents various different algorithms to manipulate the visualization of the network graph. While hierarchical network layout setups are recommended for the visualization of networks where different layers of the signal transduction are in focus, the organical network layouts can be used to group highly interconnected nodes.

Additionally the network can be modified and/or created entirely manually using yEd https://www.yworks.com/products/yfiles/documentation.

### Eliminate redundant pathways and fine tuning of the model

Parallel pathways and other combinatorial redundancies increase the solution space for Jimena, leading to the well-known problem of combinatorial explosion (e.g. known for metabolic pathway calculations, too).

Due to the mass of information on interacting biological molecules and the excessive research results it is a common bias that pathways may appear in duplicates. While some findings focus only on the general influence of a signaling pathway and hence describe it very coarse (e.g. ‘BMPs activate SMADs’) other dive in the most detailed dependencies possible (e.g. BMP6 activates BMPR1 and BMPR2, those activate SMAD1, SMAD5 as well as SMAD8, etc.). Therefore, we have to find a common model that includes the coarse but nevertheless valid findings with the detailed insights into single protein interactions and built an overall network model including all that information. Databases covering regulatory signaling networks like KEGG^22,23^, Roche or REACTOME^26^ provide an excellent starting point for the reconstruction of networks to describe divergent biological processes.

In order to achieve this, we have to eliminate those described parallel signaling pathways- and while doing so, we have to decide over the level of informational depth of our network topology. Therefore, we either consider the general model as too coarse and discard the shortcut describing this dependency or keep the coarse but nevertheless valid concept and discard the detailed signaling model considering it unnecessary in-depth.

### Network analysis: stable steady state calculation

The network model is imported into Jimena for subsequent analysis using Jimena’s build in function. Once the network is initiated in Jimena, its steady states can be calculated using the ‘SQUAD’ model. At the start of the stable steady state analysis the user must select whether to perform the discrete steady state analysis or not. Unless the network size is too large (over 100 nodes with corresponding large computation time), it is recommendable to include the discrete steady state analysis since the calculation of a complete list of steady states will speed things up resulting in a shorter required computation time. In addition, the computation time is to be set by the user. The longer the given time, the higher the probability to find continuous steady states in the network. After steady state simulation, results are shown in a popup window presenting all resulting stable steady states.

Each single state can be copied and pasted into the ‘Nodes Table’ of Jimena to map the activity of all nodes according to the stable states result onto the loaded network topology for further investigation. Alternatively, we can export the steady states into a text file for Jimena-external analysis.

In order to study the impact of important signaling molecules as well as hormones, growth factors or central receptors we can introduce an auto-activation to the respective node and hence analyze the network regarding steady states triggered by this compound. The value can also just be fixed and won’t be altered during the simulation. The auto-activation thus mimics the constant presence of this molecule and results in additional stable steady states that we can compare with the initial results without self-activation.

### Network analysis: control centrality

We used the method established by Karl and Dandekar^6^ to estimate the behavior of a network by taking into account its interconnections of the nodes and hence its susceptibility to perturbations of single nodes and vice versa its controllability at specific target proteins/nodes.

We distinguish three variations of control centralities.

Each one of the centralities is based in the convergence difference function so we start by defining that one. The convergence difference of two networks N_1_ and N_2_ is defined as:

$$\Delta \left( {N_{1} ,N_{2} } \right) = \int\limits_{{x \in \left[ {0,1} \right]^{n} }} {\mu \left( {\psi_{{N_{1} }} \left( x \right),\psi_{{N_{2} }} \left( x \right)} \right)dx}$$

The function ${\psi }_{N}\left(x\right)$ calculates the stable state of network N starting from the initial state $x$ with $n$ from the natural numbers.

The function $\mu \left(a,b\right)$ is the mean square difference of the stable states a and b.

The total centrality is the convergence difference of a network compared to a modified version of itself where one node has been deleted. Mathematically defined as (for deleting ${x}_{a}$):

$$TC\left( {x_{a} } \right) = \Delta \left( {N,N_{{del\left( {x_{a} } \right)}} } \right) = \int\limits_{{x \in \left[ {0,1} \right]^{n} }} {\mu \left( {\psi_{N} \left( x \right),\psi_{{N_{{del\left( {x_{a} } \right)}} }} \left( x \right)} \right)dx}$$

From the biological point of view this centrality measure represents a scenario where a gene modification occurs, either by knockout or overexpression or down regulation. Similarly, one could think of the use of an inhibitor molecule or any other form of directly affecting the behavior of a protein, genetic interventions (K.O., siRNA, etc.) are represented by this.

The value centrality is the cumulative convergence differences of two networks which differ only in one node value. One node is selected and assumed to be the last value in the stable state representation, then a kind of combinatorial comparison is done by comparing two exact networks (the same original network) only with the particularity that the value of the node being evaluated is fixed. Basically, the network is compared to itself while the values of the node being evaluated are changed in its whole range.

Hence, we calculate the convergence difference of a network N with a fix value a_1_ for node x_a_ with respect to the same network N but with a fix value a_2_ for the same node x_a_. Then we integrate all the convergence differences for all the possible combinations of values of the node x_a_.

Mathematically it looks like this:

$$VC\left( {x_{a} } \right) = \int\limits_{{a_{1} \in \left[ {0,1} \right]}} {\int\limits_{{a_{2} \in \left[ {0,1} \right]}} {\int\limits_{{x \in \left[ {0,1} \right]^{n - 1} }} {\mu \left( {\psi_{{N_{{\left( {x_{a} = a_{1} } \right)}} }} \left( x \right),\psi_{{N_{{\left( {x_{a} = a_{2} } \right)}} }} \left( x \right)} \right)dxda_{2} da_{1} } } }$$

For example, we have the network N:
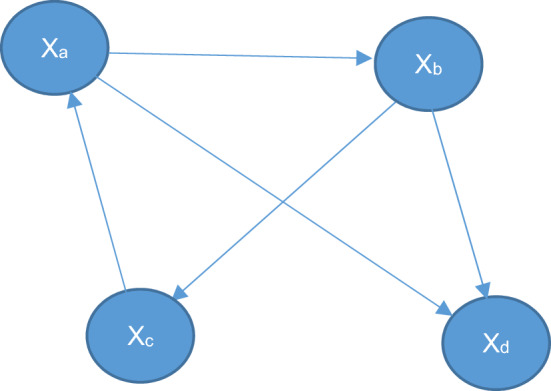


Two alternative networks (${N}_{\left({x}_{a}={a}_{1}\right)}$ and ${N}_{\left({x}_{a}={a}_{2}\right)}$) are defined based on network N. Each of those networks is exactly like N but the value of node x_a_ is fixed independently to the values a_1_ and a_2_ respectively on each of the new networks. By integrating over the whole intervals of a_1_ and a_2_ we calculate the total convergence difference for all possible combinations of a_1_ and a_2._ This total convergence difference is the value centrality of the node x_a_.

The value centrality is more related to the normal physiological conditions of the system e.g. the activation level of a receptor (“its value”). So here no gene is downregulated or severely changed but it is simply explored under normal conditions what is the role of the protein in the network. So one could think of this centrality measure as a more gradual modification of the gene expression, not a complete knockout but a slight modification. For example, to study the effects of receptors as signaling triggers in a system.

The dynamic centrality is calculated also with the help of the convergence difference function and also by modifying the original network. This time the network is modified using what is called in the paper the “split” function. This function takes one node of the network and replicates it with all its connections except one. The exact definition is given in the Supplementary material of the original paper^6^.

The function is defined as

$$\gamma_{N} \left( {x_{a} \to x_{b} } \right)$$

For example, let us use the same network N:
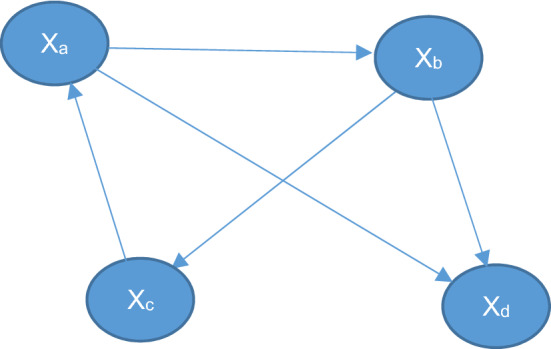


Then the split version of the network would be:
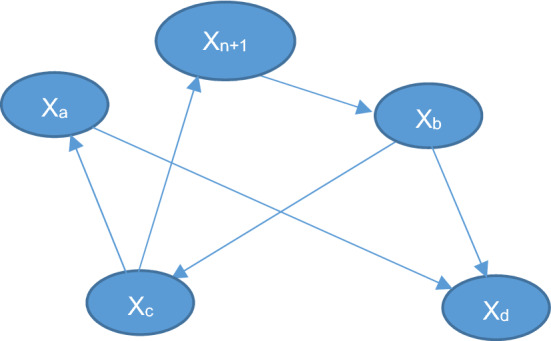


So basically a new intermediate node is created which has the same inputs from the original node which was being evaluated (in this case x_a_) and it contains only one output to the child node of the connection which is being evaluated (in this case the connection is ${x}_{a}\to {x}_{b}$ so the child node is ${x}_{b}$).

The value of the dynamic centrality of node x_a_ and connection ${x}_{a}\to {x}_{b}$ is then given by:

$$DC\left( {x_{a} ,x_{a} \to x_{b} } \right) = \mathop {min}\limits_{{y \in \left[ {0,1} \right]}} \int\limits_{{x \in \left[ {0,1} \right]^{n} }} {\mu \left( {\psi_{{\gamma_{{N\left( {x_{a} \to x_{b} } \right)_{{\left( {del\left( {x_{n + 1} \to x_{b} } \right)} \right)\left( {x_{n + 1} = y} \right)}} }} }} \left( x \right),\psi_{{\gamma_{{N\left( {x_{a} \to x_{b} } \right)_{{\left( {x_{n + 1} = y} \right)}} }} }} \left( x \right)} \right)dx}$$

where the notation ${x}_{n+1}=y$ means that ${x}_{n+1}$ has the intial value $y$.

Now, in order to compute the full dynamic centrality value of node x_a_ it is necessary to calculate this minimum value for all the outbound connections of node x_a_.

Finally, this dynamic centrality measure is a little more abstract than the other two but it has a nice biological interpretation. By eliminating a node from a network, the change induced in the network could be divided in a stable change given by the loss of the value of the node as input in the network and a more dynamic change due to the loss of the node as part of the network information flux. The dynamic centrality attempts to calculate the amount of the dynamics lost when deleting a node from a network. This is why the connections of the node are replicated on a new node and the new node is initialized with an optimal value as explained before. As for a biological interpretation of this measure we will refer to the original paper of Karl and Dandekar from 2015. In that paper the following interpretation is given *“This definition is more difficult to grasp from a biological point of view. As an example, consider the regulation of blood glucose levels in humans by insulin and other hormones such as glucagon or incretins. For a given situation of this dynamic system, the definition tries to adjust the initial insulin concentration such that the system behaves as if insulin was not involved in the regulation of blood glucose at all. If this succeeds, only the initial insulin concentration affects the systems. If it fails, which is likely in this scenario, insulin affects the system beyond its initial concentration, for example because insulin relays regulatory stimuli from other parts of the regulatory system. This is what we define as dynamic centrality “*.

So hence this centrality measure represents a pharmacological intervention regarding network effects.

### Interpretation of the results/logFC comparison/Gene expression

In order to verify the resulting values of a Jimena network simulation it is necessary to map them on experimental data (especially gene-activity data like gene expression data, e.g. micro array or RNAseq data).

**Figure 7:**
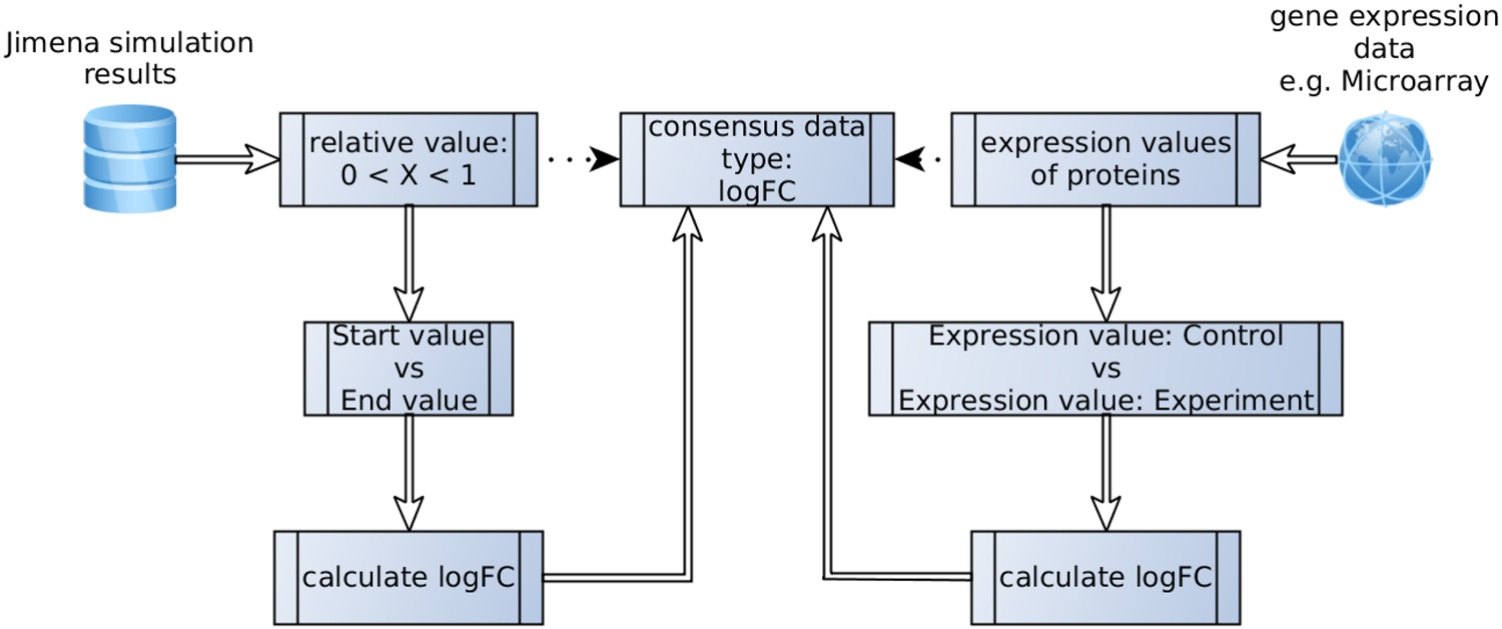
Scheme of a comparison method. Relative activity values from in silico simulations are compared to experimental gene expression data gathered from microarray results.

Since Jimena displays only values between 0.0 and 1.0 and gene expression analysis results are given in logFC with according p-values they cannot be compared straightforward. Hence, we need a method to find a consensus data type of Jimena results and gene expressions logFC values (Fig. 7).

Since logarithmic fold changes are nothing else than values describing a relative difference of one entities value in two different experimental setups (e.g. starting and ending value) and treating a Jimena-simulation as an equal experimental setup only under *in silco* conditions we assume that we can treat the resulting values of the simulation according to those in the microarray analysis: by calculating the logarithmic fold change.

Hence, we can easily compare in silico results with their experimental counterpart and conclude the biological validity of the reconstructed model network (Table 2).

**Table 2:** Example comparison of simulation values versus gene expression data^1^.

Protein	Jimena		Gene Expression	
	Start	End	logFC	logFC
A	0.100	0.500	0.699	0.750
B	0.001	0.100	2.000	2.500
C	0.500	0.090	-0.745	-1.000
D	0.900	0.100	-0.954	-1.000

^1^The logFC value from Jimena was calculated as the logarithmic value of a nodes ratio from its initial value divided by its value at the end of the simulation and (log(xend/xstart)). Thus both different results become comparable.

### Dynamical simulation

Based on the results of the steady states analysis we can initialize a dynamic simulation to test possible experimental approaches in order to alter the networks behavior. Each steady state in theory stands for one cellular state existent in nature and thus can be termed a biological function. After identification of a suited starting steady state we can load this state as an initial starting point for our simulation.

The preset of 50 arbitrary time units simulation time, a step size of dt = 0.05 as well as a maximum computing speed can be changed according to required values. While most of the network simulations level out between 20 and 30 arbitrary time units (ATU) sometimes a longer time is advantageous to monitor the overall reactions of the network. The time units should be sufficiently long such that the network can converge to its steady state. Numerically this can be checked if the time curses of the values of the nodes are each a horizontal line at the end of the simulation (after the transient where values vary). While the computing speed doesn’t change the results of the simulation, the step size dt is the most crucial variable and can change the complete numerical calculation results. Nevertheless, we achieved excellent results using the preset value of dt = 0.05, but we don’t recommend bigger values since they may result in coarser and more imprecise simulation results. The reason for the behavior is that Jimena uses an explicit solver (forth order Runge–Kutta^11^) to calculate the solutions to the ODE systems. These explicit solvers suffer from numerical instabilities if the step size (time discretization) is chosen too coarse. Please see the Supplementary material “Mathematical Appendix” for further information.

The dynamic simulation can be monitored while simulating via the Jimena built in charts window in shape of a customizable graph view as well as on the Jimena main window displaying the network topology. White nodes depict inactive nodes while an activation is represented by a grey color. The darker the grey, the higher the activity value of the respective node. While the charts window allows for an export of the result to text file, the main window only acts as an illustration of the networks behavior.

Subsequently, the resulting simulation can be compared with the steady state results in order to map it on systemic function and giving an indication how to treat the model to change its determined path.

Additionally, to auto-activating loops, Jimena provides a perturbation function by which we can alter the activity value of selected nodes over a defined time. Thus, we can simulate in vitro approaches using different knock-down or stimulating agents.

### Technical comments

These are given in detail on our public website.

https://www.biozentrum.uni-wuerzburg.de/bioinfo/computing/jimenae/. We give as Supplementary material also all Matlab scripts and the Jimena program (file: jimenaProgramScripts.zip).

We provide detailed information online on how to achieve:

### Data driven modelling

Comparison of the semiquantitative modelling with the observed experimental data allows to normalize the time scale, e.g. according to the hours and time-scales the experiment did take the time scale can be given accurately. Similarly, comparison to the observed experimental data allows to find out how strong maximal activation of a “node” in the model is, e.g. according to protein phosphorylation data.

Alternatively, if the function of a particular node is known by some source of information, these values can be plotted directly instead of approximating by Jimena and compared to the activation profile of the remaining network. This is done by a Matlab script.

### Centrality calculations

A step size routine for calculation of centralities has been implemented in the software. In cases where convergence is difficult to achieve, users can vary step-size, in particular smaller step size to achieve convergence despite the complex solution space. The time interval setting is crucial regarding the time-scale of the computation, because the simulation result can agree with experimental data only under this condition that a proper time interval is specified. We use 0.1 as the default time interval (“dt”) in the software, which works sufficiently fast and reliable for most centrality computations. However, 0.01 will be a better choice to obtain a more precise result if more computational power and time is available.

### Online read-out of values

Steady states and gene lists can be exported from JimenaE into plain-text data files. Together with the generated Matlab scripts, researchers can proceed to carry out different analysis in Matlab. In addition, the two scripts we offered in the package allow to plot the simulation curves directly in Matlab.

### Programming editor

Eclipse set up is easily possible, including the commented Jimena program. The source code package allowing a programmer to develop the code further in eclipse is made available.

### Cell–cell interaction models

As an example, T-cell maturation can easily be modeled in a multi-compartment model using Jimena. The complete and detailed model (“T-cell-concept”) is given as graphml,file for network set-up and refinement, as well as tiff and png file. Moreover, a simplified model, which was reduced to the basic mechanisms of regulation inside a single T-cell is provided within the supplement. The simplified model is also given in graphml.-, tiff-, and png.-format.

On these a dynamical simulation can be build. This requires then detailed data on the kinetics for data driven modeling, however, the basic topology given can already now be read-in by Jimena and edited as needed by yEd graph editor.

### Ethics declarations

We confirm that our research does not directly involve neither human cells / tissues nor animal subjects, or plants. This is a software and data analysis manuscript. Hence no ethics declarations need to be added to our manuscript.

## Supplementary Information

Supplementary Information.

## Data Availability

All data are contained in the submitted files and figures and its supplementary material and the deposited transcriptome data. Jimena is available for download and with tutorial at https://www.biozentrum.uni-wuerzburg.de/bioinfo/computing/jimenae. We give here also the database repository accessions for all large-scale data sets used in our study. Data sets: Analysis of mesenchymal stem cells (MSCs): GSE9451; GSE18394; GSE19664; GSE 28205; GSE42352, GSE10192. Arabidopsis, hormonal stimuli of salicylic acid: GSE3984; influence of cytokinins: GSE5520; Dendritic cells: GSE69723.

## References

[1] Sideris, T. C. *Ordinary differential equations and dynamical systems* (Atlantis Press, 2013).

[2] Di Cara A, Garg A, de Micheli G, Xenarios I, Mendoza L (2007). Dynamic simulation of regulatory networks using SQUAD. BMC Bioinform..

[3] Müssel C, Hopfensitz M, Kestler HA (2010). BoolNet–an R package for generation, reconstruction and analysis of Boolean networks. Bioinformatics (Oxford, England).

[4] Hoops S (2006). COPASI–a complex pathway simulator. Bioinformatics (Oxford, England).

[5] Klamt S, Saez-Rodriguez J, Gilles ED (2007). Structural and functional analysis of cellular networks with Cell NetAnalyzer. BMC Syst. Biol..

[6] Karl S, Dandekar T (2015). Convergence behaviour and control in non-linear biological networks. Sci. Rep..

[7] Czakai K (2017). Influence of Platelet-rich Plasma on the immune response of human monocyte-derived dendritic cells and macrophages stimulated with *Aspergillus fumigatus*. Int. J. Med. Microbiol.: IJMM.

[8] Osmanoglu Ö, Shams S, Dandekar T, Naseem M (2021). Modeling immune dynamics in plants using JIMENA-package. Methods Mol. Biol. (Clifton, N.J.).

[9] Krumsiek J, Pölsterl S, Wittmann DM, Theis FJ (2010). Odefy–from discrete to continuous models. BMC Bioinform..

[10] Mendoza L, Xenarios I (2006). A method for the generation of standardized qualitative dynamical systems of regulatory networks. Theor. Biol. Med. Model..

[11] Karl S, Dandekar T (2013). Jimena: Efficient computing and system state identification for genetic regulatory networks. BMC Bioinform..

[12] Batt G (2012). Genetic network analyzer: A tool for the qualitative modeling and simulation of bacterial regulatory networks. Methods Mol. Biol. (Clifton, N.J.).

[13] Thiele S, von Kamp A, Bekiaris PS, Schneider P, Klamt S (2021). CNApy: A cell netanalyzer GUI in python for analyzing and designing metabolic networks. Bioinformatics (Oxford, England).

[14] Schwarz R (2007). Integrated network reconstruction, visualization and analysis using YANAsquare. BMC Bioinform..

[15] Breitenbach T, Liang C, Beyersdorf N, Dandekar T (2019). Analyzing pharmacological intervention points: A method to calculate external stimuli to switch between steady states in regulatory networks. PLoS Comput. Biol..

[16] Breitenbach T, Lorenz K, Dandekar T (2019). How to steer and control ERK and the ERK signaling cascade exemplified by looking at cardiac insufficiency. Int. J. Mol. Sci..

[17] Kerkhofs J, Roberts SJ, Luyten FP, van Oosterwyck H, Geris L (2012). Relating the chondrocyte gene network to growth plate morphology: From genes to phenotype. PLoS ONE.

[18] Naseem M (2012). Integrated systems view on networking by hormones in Arabidopsis immunity reveals multiple crosstalk for cytokinin. Plant Cell.

[19] Setty Y (2014). In-silico models of stem cell and developmental systems. Theor. Biol. Med. Model..

[20] Bian Q, Cahan P (2016). Computational tools for stem cell biology. Trends Biotechnol..

[21] Raue A (2015). Data2Dynamics: A modeling environment tailored to parameter estimation in dynamical systems. Bioinformatics (Oxford, England).

[22] Kanehisa M (2014). Data, information, knowledge and principle: Back to metabolism in KEGG. Nucl. Acids Res..

[23] Kanehisa M, Goto S (2000). KEGG: Kyoto encyclopedia of genes and genomes. Nucl. Acids Res..

[24] Franceschini A (2013). STRING v.91: Protein-protein interaction networks, with increased coverage and integration. Nucl. Acids Res..

[25] Szklarczyk D (2015). STRING v10: Protein-protein interaction networks, integrated over the tree of life. Nucl. Acids Res..

[26] Gillespie M (2021). The reactome pathway knowledgebase 2022. Nucl. Acids Res..

