# Supplementary material for: Software JimenaE allows efficient dynamic simulations of Boolean networks, centrality and system state analysis
Source: Sci Rep. 2023 Feb 1;13:1855. doi: 10.1038/s41598-022-27098-7 (PMC9892028; doi:10.1038/s41598-022-27098-7)
Supplement: Supplementary file 1 — Supplementary Information. [file 41598_2022_27098_MOESM1_ESM.zip › All-Supplements/supplement_Tables_Appx_revise-Fi.pdf]

Supplemental materials: Software JimenaE allows efficient dynamic simulations of Boolean networks, centrality and system state analysis

Martin Kaltdorf[§,1], Tim Breitenbach[§,1,2], Stefan Karl[§,1], Maximilian Fuchs[§,1], David Komla Kessie[3], Eric Psota[4], Martina Prelog[4], Edita Sharukanyan[1], Regina Ebert[4], Franz Jakob[4], Gudrun Dandekar[5], Muhammad Naseem[1,6], Chunguang Liang[1,*], Thomas Dandekar[1,*]

[1]Department of Bioinformatics, Biocenter, University of Würzburg, Am Hubland, 97074 Würzburg, Germany; [2]Institut für Mathematik und Informatik, University of Würzburg, Am Hubland, 97074 Würzburg, Germany;[3]Department of Microbiology, Biocenter, University of Würzburg, Am Hubland, 97074 Würzburg, Germany; [4]University Hospital Würzburg, Pediatric Rheumatology/Special Immunology, Josef-Schneider-Str. 2, 97080 Würzburg, Germany;[5]Orthopedic Center for Musculoskeletal Research, University of Würzburg, Friedrich-Bergius Ring 15, 97076 Würzburg, Germany; [6]Chair of Tissue Engineering and Regenerative Medicine, University Hospital Würzburg, Röntgenring 11, 97070 Würzburg, Germany; [7]Fraunhofer Institute for Silicate Research (ISC), Translational Center Regenerative Therapies, Röntgenring 11, Würzburg 97070, Germany; [8]College of Natural and Health Sciences, Department of Life and Environmental Sciences, Zayed University, Abu Dhabi; *Correspondence: dandekar@biozentrum.uni-wuerzburg.de; liang@biozentrum.uni-wuerzburg.de; [§]These authors have equally contributed to this work

Contents:

Analyzing biological networks with Jimena:

Rapid identification of system states (example: mesenchymal stromal cell plasticity and differentiation)

Modelling dynamics of cell interaction (example: Arabidopsis infected by *Pst* DC3000)

Modelling types of network control (example: immune cells sense fungi)

Supplementary Data Files to set-up and analyze the three above example networks:

"All-network-files-supplement" (AT Arabidopsis, DC dendritic cells, MSC mesenchmyl stromal cells: 12 files in total) for each network we provide: (i) the graphml file for read-in to Jimena (MSC_Model.graphml, AT_Athal.graphml, DC_Afu_Model.graphml); (ii) the excel table giving references and wiring of the network (MSC_RefList, AT_RefList, DC_RefList); (iii) the stable system states analysis done by Jimena (MSC_SSS.txt, AT_SSS.txt, DC_SSS.txt). (iv) all three centrality values calculated by Jimena (MSC_CC.xlsx, AT_CC.xlsx, DC_CC.xlsx)

Detailed further analysis of the three biological application examples:

Part I --Analyzing mesenchymal stem cell (MSC) differentiation using Jimena

Part II --Analyzing checks and balance mechanisms in plant *A.thaliana* (AT) signaling networks

Part III --Analyzing immune response network control in murine dendritic cells (DCs)

Mathematical appendix (Jimena in depth)

Analyzing biological networks with Jimena:

Rapid identification of system states (example I: mesenchymal stromal cell plasticity and differentiation)

Using mathematical models of biological systems enables breaking down highly complex molecular processes into well-arranged and comprehensible parts.

In particular, the Jimena modelling suite is also fully capable of identifying different system states. Stable states are states where the network reaches an equilibrium without external stimuli or input. For instance, in cancer cells either the proliferative or the apoptotic pathways are more active and hence, this leads then two possible system states: one drives tumor formation further, the other one starts to eliminate the tumor cell.

With the MSCs the challenge is that from different system states different pathways for further differentiation are activated [1]. Each of this system states is accessible for MSCs. Due to this involvement of many different pathways the network topology of MSCs is even more sophisticated than the two previous examples and consists of 161 nodes and 347 edges resulting in a network density of 2.15 (Figure 1 in paper). Jimenas' steady state analysis calculated 9 stable steady states (Figure 2). A steady state of a network is where the expression levels do not change any more, see the Mathematical Appendix of the supplement. Again, these stable steady states calculated by the Jimena package reveal fascinating cell biology. Here, we see how MSCs can differentiate into three more specialized cell types by changing its system state: The stable steady states can be divided into three groups (Table S1): (1) the first group of stable steady states (stable steady states 8, 9) can be characterized by activity of adipogenic transcription factors PPARG (Peroxisome Proliferator Activated Receptor Gamma) and CEBPA (CCAAT Enhancer Binding Protein Alpha) while all signature proteins of other differentiating fates are downregulated. (2) The second group of stable steady states (stable steady states 1-4) presents a strong upregulation of osteogenesis in general triggered by multiple osteogenic marker genes (ALPL (Tissue Non-Specific Alkaline Phosphatase), RUNX2 (RUNX Family Transcription Factor 2), CTNNB1 (β-Catenin)). While

the activity of adipogenic factors is still high, chondrogenic marker genes (ACAN (Aggrecan) and SOX9 (SRY-Box Transcription Factor 9)) show no significant upregulation. (3) The third group of stable steady states (5-7) illustrates contrasting activity patterns compared to the first group. With upregulation of chondrogenesis initiated by ACAN and SOX9 and intermediate activity of some osteogenic markers ALPL and RUNX2, all adipogenic factors are completely inactivated. Although ALPL and RUNX2 show high activity the overall signal is not sufficient to trigger the activation of osteogenesis. Both groups (2 and 3) display an inhomogeneous behavior regarding cell cycle and apoptosis – antagonistic mechanisms which can mutually be observed here. These results illustrate the antagonistic behavior of chondrogenic and adipogenic developmental processes initiated by the activity of the wnt-signaling pathway signalling (details in supplemental file MSC_SSS.txt) This shows again that cell biological processes, in this case cell cycle, programmed cell death and furthermore the differentiation exploit and perpetuate different network states.

Applying control centrality analysis by Jimena on MSC differentiation we can identify four well known candidates with strong controlling influence on cell fate (RUNX2, OSX/SP7, SOX9) and cell cycle (TP53) as well as PPRG for adipogenic differentiation (Fig. 2 in paper). Those proteins are important to MSC differentiation leading to a deprivation of differentiation capabilities in case of one of their deprivation. Furthermore, the analysis revealed a primary function of SMAD2 and 3 as well as TGFB-receptors I and II in course of cell fate control of MSC.

Validation by gene expression data sets supports this (Table S1): We summarized the analysis of six different experimental datasets gathered from gene expression omnibus [2] in eight different comparisons (GEO2R) for osteogenic, chondrogenic and adipogenic differentiation by calculating the mean over the activation of all key genes to allow for an easy comparison with calculated *in silico* results. The GEO data confirm that ALPL and RUNX2 have a substantial role in osteogenesis. Although ACAN is upregulated, the inactivation of SOX9 supports the assumption that chondrogenic mechanisms are shut down during osteogenic

differentiation. Nevertheless, the upregulation of PPARG and a subtle activity of CEBPA suggest at least some influence of adipogenic factors during osteogenesis. Activation of ACAN and SOX9 during chondrogenesis are as expected [3]. Nevertheless, RUNX2 and ALPL seem contradictory regulated to adipogenic determination [4]. Adipogenesis is obviously influenced and sustained by CEBPA and PPARG, which is in line with all state-of-the-art biological data published as yet.

Chondrogenesis and osteogenesis partly overlap (shared network proteins; detailed separation by surface markers such as Sclerostin and YAP/TAZ) while adipogenesis is a more distinct differentiation path. This can be studied in detail for MSCs examining control centrality (Fig. S1 centrality analysis plot; Table S2, gives detailed centrality values for key proteins).

Stromal cell plasticity is enhanced by autocrine loops

Figure 3 (paper) illustrates the plasticity of stromal cells and how this can be studied using Jimena. The histogram illustrates the number of equilibria states of the MSC-network model in dependency of a variable number of autocrine activating stimuli.

Besides the calculation of stable steady states, i.e. where the levels of the nodes do not change any more, and diverse control centralities, Jimena comes with functions to readout and even to modify the analysis' algorithm to suit versatile needs in a fully replicable manner and thus provides for a complete analysis suite for the test of directed interaction networks. This is instrumental to better understand the design of a specific biological network. Thus, the topology and regulatory power changes a lot with or without auto-stimulatory loops. Such loops occur in many differentiation processes [5] either at the core of differentiation regulation, e.g. transcription factors CEBPA and PPARG activate each other in our network [6] or in form of permanent presence of growth and transcription factors (e.g. isoforms of TGFB, BMP, IGF, etc.) that especially occur in pathological, (pre)cancer states [7]. The fragility of cell division and tendencies to increase cancer proneness with every division has also been investigated and stressed by Tomasetti et al. (2017) [8].

Jimena can model the high plasticity of pluri- or multipotent cells with many system states in this way. For MSC plasticity the analysis is as follows:
Analyzing the network topology, we applied a varying number of auto-stimulatory loops (ALs) for well know transcription factors and receptors that lead to continuous self-activation of a specific node. We could show that the plasticity and hence the possible stable system states increases exponentially with the number of such auto-stimulatory loops (4 ALS: 67 SSs; 10 ALs: >1800 SSs). The high number of SSs is instrumental for the multipotency of mesenchymal stromal cells. As Jimena allows to directly simulate and verify the control of differentiation in MSC, we can verify that differentiation stepwise removes such loops and leaves only fewer differentiation routes open as shown above with the three classes of system states available for the MSC to differentiate into adipogenic, chondrogenic and osteogenic cells. Jimena show by these simulations that activation of growth factors pave the way for a multipotent to more differentiated state shift of the cell into any of the three more differentiated cell types. Growth factors are vital for reprogramming cells, in particular to achieve the high plasticity state in mesenchymal stromal cells ("multipotency").

Auto-secretion [9] is also observed for cells during embryology, in normal, aging [10] and senescent cells [11]. This allows multiple cellular states and high plasticity and this is advantageously and easily simulated and analyzed with Jimena. Importantly, also the high plasticity of the cancer cell state is concomitant with dedifferentiation and malignity [12] and can also be analyzed with our software.

Gene Expression Comparisons validate this, for instance the data set GSE9451 where we see in group1 the specific differentiation into osteoblasts, chondrocytes, adipocytes compared to the starting group2, the MSCs.

Thus, we show in this third part of results the differentiation path of stromal cells to generate osteoblasts: Simulations show that we have a highly flexible "multipotent" state if we have

many auto-secretory loops or growth factor stimulated loops. In such a case a precursor cell can adapt into many differing states. The number of which decreases along the different stages of lineage commitment from pluripotency over multipotency to the final commitment and maturation:

(i) If we look at the simulation results and compare them with the gene expression data, there are basic multipotency inducing factors present initially. If we consider these, then there are only 20 different system states. This we assume corresponds to the basic MSC state, a number of states which allows plasticity, but not the high number of different states accessible to multipotent mesenchymal stroma cells.

(ii) If specific differentiation factors are secreted, the system can be driven towards three directions: Adipocyte, chondrocyte and osteoblast development. There is a specific combination of transcription factors needed for each differentiation direction to stimulate the specific receptor(s) and transcription factors (Table S1). (iii) Finally, the cells reach their more defined differentiation state as either osteoblasts, chondrocytes or adipocytes, respectively. Jimena shows, in these more restricted plasticity state there are only less different cellular states available for each of them with different pathway activities. Overall 9 stable system states could be identified. There are 4 states for osteoblast differentiation (1,2,3,4), 3 states for chondrocyte differentiation (5,6,7) and 2 for adipocyte differentiation (8,9). The detailed states are shown in Figure 2. Jimena verifies by this system state stability analysis that these differentiation pathways have here been selected and optimized to stabilize these cellular system states.

Regarding validation we show here by gene expression data sets and their analysis that these data from transcriptome analysis indeed match the simulation results and support these conclusions and routes of differentiation (see Figures in supplement).

Modelling dynamics of cell interaction (2nd example: Arabidopsis infected by *Pst* DC3000)

Pseudomonas syringae pv. *tomato* DC3000 is a bacterial plant pathogen that threatens the cultivation of many different major crop plants worldwide, is an important model organism to examine the immune system of plants. Our model network illustrates the interplay between the pathogenic elicitors and the defensive response of *A.thaliana* (Figure 4A, paper). The network architecture consists of 104 regulatory components, in network biology often referred as nodes, connected by 164 interactions (generally described as edges). The edges-to-nodes ratio gives a valid estimate of the network structure: biological, scale-free interaction network-densities with lower density values imply low adaptation capabilities, higher values on the other hand make a network unstable. The optimal range of 1.4 - 2.75 [13] has proven to provide the best ratio between controllability and vulnerability against external disturbances – a ratio found prevalently in biological systems.

Moreover, we modeled the complex signaling processes between various plant hormones [14–16]. We show here that there are only three components among the previously selected important nodes to show a high controlling influence (value centrality of more than 10^{-3}) on the network: Jasmonic acid, bacterial pathogen *Pseudomonas syringae* (the specific strain investigated is *Pst* DC3000 *TGA*; interactions in Fig. 4B, paper). The network centralities as calculated by Jimena and examined in Fig. 5A (paper) control system stability.

We identified two stable cellular states (SSS, Figure 4B), i.e. states of the cellular network where the pathways and activation of corresponding genes have a self-stabilizing pattern of gene expression (“steady state”), using the built-in feature of Jimena: both of them show activation of *Pst* DC3000. While stable steady states No 1 can be stated as a complete inactive state, stable steady states No 2 shows activation of Jasmonic Acid (JA) and Auxins [16].

Using publicly available gene expression data (GSE3984 and GSE5520) as well as targeted experiments [17] for validation, we proved that using our *in silico* approach can simulate the real *in vivo* behavior of a plants immune reaction in close approximations. In particular, using

Jimena all system responses of the plant immunity are now well modeled dynamically and quantified regarding relative intensities and series of events: After pattern recognition and pattern triggered immunity (PTI), there come the plant elicitors and as a host response the elicitor triggered immunity (ETI). The basic zig-zag model [18] is transformed into two waves of response measures, fine-tuned against each other and consecutive (see Figure 5).

An important result is that pathogen *Pst* DC3000 by evolutionary adaptation has direct control of the network as a major and direct trigger of the immune response. Moreover, TGA, the transcription factor, is here singled out as an important factor with direct control. Finally, Jasmonic Acid proofs instrumental for modulating the network. The counter player of the hormone orchestrating response against pathogen *Pst* DC3000, i.e. salicylic acid, would not help against the pathogen: As the check and balance model shows (Fig. S2), the value centrality, direct centrality and total centrality values are low here for Salicylic acid. Moreover, *Pst* DC3000 inhibits cytokines, the only way to activate salicylic acid as well as the pattern recognition node (see Fig. S2). Furthermore we can show that Jasmonic acid and transcription factor TGA but not *Pst* DC3000 are dynamically involved in stimulating core signal transduction pathways (direct centrality of more than10^{-3}), confirming their central role in the plants' immune response to pathogenic infections [17,19]. Centralities for dominating nodes are given in Table S3. Fig. 5B illustrates the matching system states dominated by specific nodes such as *PstDC 3000*.

The dynamic simulation demonstrates the complex dynamic interplay emerging from such a network topology and the nontrivial intermediate states between stable states (Figure 5C): PR1, a primary resistance marker in *A.thaliana* [15], shows two distinct peaks in activity due to infection by *Pst* DC3000 appropriately illustrating the complex struggle between host and pathogen. Using *in silico* simulation based on Jimena, we can easily simulate the influence of different pathogenic mutants without the need for extensive experimental setups and work.

We show here as a new insight the network control involved. Jimena allows to model VC (value centrality), i.e. direct network control, DC (dynamic centrality, i.e. impact and control via other

network nodes and TC (total centrality) as a combination of both; Fig. 5A; detailed values Table S3).

Modelling types of network control (example: immune cells sense fungi)

Human dendritic cells play an important role in the mediation of T-cell activity due to pathogenic infection and are thus a central component of the human adaptive immune system. We investigated their action against *Aspergillus fumigatus* in human infection [20]. Since especially patients already suffering from immunodeficiency show high susceptibility against infectious aspergillosis, the development of new, well-tolerated therapeutic strategies is vital. Extending our earlier study, we can now use the systems analysis capabilities of Jimena to study this system further and learn more about the network control of the different nodes, another feature of Jimena. Network control is critical to mediate fungal defense response by human dendritic cells. There are 179 interactions and 86 nodes (see Fig. 6, paper). The network density of 2.081 is significantly more complex than in our *A.thaliana* model and thus results in a higher number of steady states. Their classification is immunologically relevant:

Our detailed analysis of network control and centrality in murine dendritic cells using Jimena (Fig. S3) identifies chemokine 5 ligand (CCL5), Dectin-1, IL-1, IL-6, toll-like receptor 2 (TLR2) and TLR4 as equally important and involved in modulating the signaling cascades (centrality analysis of these nodes is given in Table S4).

Table S5 shows that there exist different groups of steady states regarding maturation markers such as CD80, CD83 and CD86 while in the other groups these markers are less active. Regarding the network, each single group shows significant internal differences in platelet and IL-6 only, highlighting the direct control of IL-6 activity by platelet factors. This difference is vital for a better healing process since interleukins like IL-6 induces inflammatory responses in infected tissue [20].

Jimena allows for these dendritic cells to reveal a specific role of IL-1 (strong total centrality and direct centrality, low value centrality) in activating immune response and of IL-6 (strong total centrality and direct centrality, low value centrality) in mitigating inflammation in this system for immune responses against fungi. This is different from the role of IL-6 in some other infections or in autoimmunity and the role of IL-6 as a key cytokine for stimulation of differentiation of B cells into plasma cells). We use again gene expression data (dateset GSE69723) for demonstration. In our model, we see that the hub nodes NFκB (betweenness centrality value of 1.00, total centrality of $7,97^{-02}$ and value centrality of$1,17^{-02}$) and Tumor-necrosis-factor alpha (abbreviation: TNF) (betweenness centrality value of 0.65, total centrality of $3,45^{-02}$ and value centrality of$1,32^{-02}$) have high total centrality and value centrality values, channeling the immune response. Modulatory crosstalk by bystander nodes is both there for pro-inflammatory and anti-inflammatory stimuli (e.g. cytokines), but this is weaker than the control by external stimuli (e.g. platelets).

Validation with gene expression data (GEO data series GSE69723) confirmed that the network model predicted well the expression changes compared to the experimental results (see [20]). Another fundamental pathway of the human immune system, T-cell maturation, can also be modeled and analyzed using Jimena (Fig. S4).

Discussion of the three biological application examples

The analysis results demonstrate the versatile opportunities arising and biological insights resulting from the application of Jimena: We could show that the value control centrality acts as a valid method to identify major controlling nodes of a network that can be used to alter its overall behavior when changed (strong control centrality with values between 10^{-03} and 1.0, Fig 4 paper and Fig S5). Furthermore, we verified that the dynamical control centrality states a solid approach to identify central signaling pathways by calculating their nodes' relaying function (Fig 4 paper and Fig S5). The control centrality proved to be superior compared to previous centrality measures of other software platforms regarding its prediction of regulatory

influence on the network topology (e.g. Cytoscape). We were able to demonstrate that the calculated stable steady states realistically represent cellular states of defined function: (1) for our *A.thaliana* model we identified one state of permanent *Pst* DC3000 presence and two states without *Pst* DC3000 where one illustrates an upregulated immune function while the other can be interpreted as a naïve state without any activity of immunity related components. (2) Furthermore, we could calculate various steady states of human dendritic cells under influence of infectious aspergillosis describing possible conditions of a dendritic cell: unbiased cellular function, cellular function under influence of platelets and the impact of *A.fumigatus* on the regulation of relevant molecular components like the maturation markers of dendritic cells that trigger B-cell maturation CD80, CD83 and CD86. Finally, (3) we examined major interactions that establish the differentiation regulation of mesenchymal stromal cells. The analysis of the network concluded in three distinct groups of activation patterns illustrating the possibilities the commonly known differentiation mechanisms can occupy. The key factors for adipogenesis (CEBPA, PPARG), chondrogenesis (SOX9, ACAN) and osteogenesis (ALPL, RUNX2) indicate the prevailing condition. The analysis shows the existence of distinguishable steady states for each differentiation pathway: steady states 1-4 for osteogenesis, steady states 5-7 for chondrogenesis with osteogenic influence as well as adipogenesis (steady state 8 and 9) consistent with data on committed cellular system states for each of the three differentiation types, e.g. chondrocytes (reviewed in [21]). Gene expression analysis of different GEO datasets confirm the general significance of the simulated data showing similar gene activation patterns (osteogenesis with chondrogenic influence, chondrogenesis with osteogenic influence and adipogenesis).

Since the isolation of embryonic stem cells (ESCs) in 1981, a question of intense focus has been, 'What are the molecular mechanisms by which pluripotent stem cells (PSCs) maintain multilineage potential indefinitely?' OMICs techniques have played a central role in answering this question and have revealed previously unanticipated complexity in the regulation of pluripotency. As a first step towards understanding the molecular basis of pluripotency, the

transcriptional profile of ESCs, and its relationship to that of adult stem cells was defined. Subsequently, the predecessor to ChIP-Seq, ChIP-chip, was used to reconstruct to transcriptional regulatory network of core pluripotency transcription factors (TFs) in human ESCs, uncovering an autoregulatory loop that helps to maintain the pluripotent state by buffering against transient downregulation of any single pluripotency transcription factor. This network motif was found by ChIP-chip to be conserved in mouse ESCs. To further define the pluripotency regulatory network at the level of genomic regulatory elements, the Stamatoyannopoulos group paired distal DNase hypersensitivities (DHSs) with target promoters of pluripotency-specific TFs such as KLF4, SOX2, and OCT4 [22].

Jimena is well-suited to model different systems biological effects gaining recent attention in pluripotent stem cells, for instance to model how TGFß signaling maintains pluripotency of human naïve pluripotent stem cells [22] or to model the human epiblast and yolk sac interaction in an *in vitro* stem cell model [23].

Supplementary Data Files to set-up modelling environment:

For the three examples we give the data-files to set them up and analyze them:

(i) the corresponding network files with references in excel format: MSC_RefList, AT_RefList, DC_RefList,

(ii) the calculated centrality values as excel files: MSC_CC.xlsx, AT_CC.xlsx, DC_CC.xlsx,

(iii) the stable system states plus the values then each node has, as txt files: MSC_SSS.txt, AT_SSS.txt, DC_SSS.txt.

(iv) Finally, the Jimena readable files for the networks examined are given in graphml format: MSC_Model.graphml, AT_Athal.graphml, DC_Afu_Model.graphml,

Detailed further analysis of the three application examples:

Part I --Analyzing mesenchymal stem cell differentiation using Jimena

In further analysis using Jimena for investigating this signaling network, the fundamental behavior of the MSC (mesenchymal stem cells, also called stroma cells) differentiation can be explained by the opposing characteristics of a core network around consisting of three prime transcription factors: SOX9 for chondrogenesis, PPARG for adipogenesis and RUNX2 for osteogenesis. In presence of either one of them the activity of the other two is inhibited [15–18]. By the application of Jimena we now can further analyze the dependencies and controlling influences in the regulatory mechanisms that lead to the directed activation of only one of those developmental processes. The MSC network was established by iterative refinement according to expert knowledge (TD, FJ, RE, MK), literature and experimental results. For validation gene expression data from different laboratories were considered (GEO data sets are indicated). In particular, we want to compare the MSC and differentiation pathways (healthy differentiation and adaptation) with tumor growth in the bone niche. As a basis for this we now analyze here the MSC differentiation pathways and observe also a high MSC plasticity which is partly mirrored also by tumor cells, in particular tumor stem-cells, and again autocrine loops could be an important mediator for this (not followed up here, but in other more cancer-minded publications from us).

Based on the Value Control Centrality the factors with primary function on cell fate control of MSC such as transcription factors SMAD2 and 3 and their upstream modulators TGFB-receptors I and II revealed also a strong impairing influence in case of loss-of-function of those nodes.

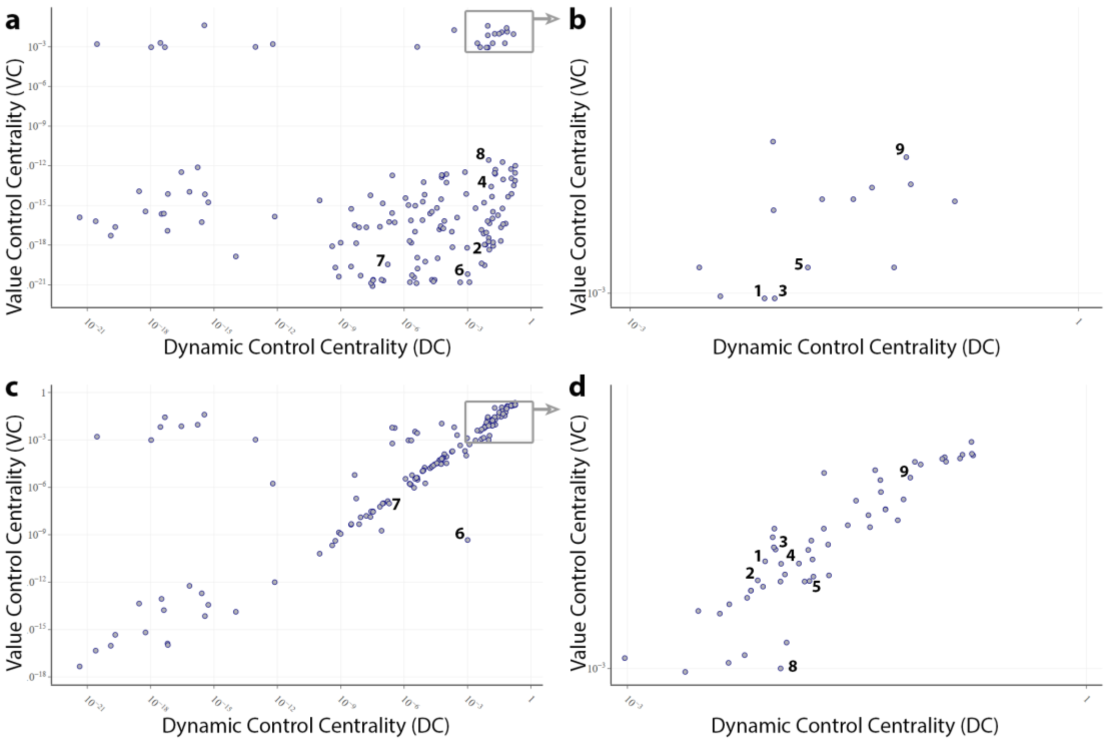

Supplementary Figure S1: Control centrality analysis using Jimena in a mesenchymal stem cell network identifies control type of key transcription factors. Control Centrality values accentuating the controllability, susceptibility and signal transducing importance of the network nodes. (A+B) VC (steerability) plotted against DC (signal transduction) and (C+D) TC (susceptibility) plotted against DC highlight the role of each node and allow for the identification of important controlling influences (Control Centrality value > 10^{-3}). Dominating components are marked by numbers: 1. Runt-related transcription factor 2 (**RUNX2**); 2. Alkaline phosphatase, tissue non-specific isozyme (**ALPL**); 3. Transcription factor SP7 (**SP7**); 4. Catenin beta-1 (**CTNNB1**); 5. Transcription factor SOX9 (**SOX9**); 6. Aggrecan (**ACAN**); 7.CCAAT/enhancer-binding protein alpha (**CEBPA**); 8. Peroxisome proliferator-activated receptor gamma (**PPARG**); 9.Tumor protein 53 (**TP53**).

Table S1: Comparison of experimental Mesenchymal stem cell results (GEO datasets) and calculated Stable Steady States

network nodes / GEO datasets	chondrogenesis					osteogenesis					adipogenesis			
	GSE9451	GSE18394	GSE19664	Simulation		GSE9451	GSE28205	GSE42352	Simulation		GSE9451	GSE10192	Simulation	
chondrogenesis factors				→					↓				↓	
ACAN	↑	↑	na	↑	✓	↑	na	na	↓	✗	↑	↑	↓	✗
SOX9	↑	na	↑	→	–	↓	↓	↓	↓	✓	na	na	↓	?
osteogenesis factors				↓					↑				↓	
ALPL	na	↑	na	↓	✗	↑	na	↑	↓	✗	↑	↓	↓	–
RUNX2	na	na	↓	↓	✓	↑	na	→	↓	✗	↓	na	↓	✓
CTNNB1	↑	na	na	↓	✗	↑	na	↓	↓	–	↓	↓	↓	✓
adipogenesis factors				↓					↑				↑	
CEBPa	na	na	na	↓	?	↑	↓	↑	↑	✓	↑	↑	↑	✓
PPARG	↓	↓	na	↓	✓	na	↑	↑	↑	✓	↑	↑	↑	✓
apoptosis/cellcycle fact				↑					↓				↓	
MYC	na	na	na	↑	?	na	na	↑	↑	✓	↑	↓	↓	–
TP53	na	↓	na	↓	✓	na	na	→	↓	–	↓	na	↓	✓
CDKN1A	na	na	↓	↓	✓	na	na	↑	↓	✗	na	↓	↓	✓
CFLAR	↓	↓	→	↓	✓	↑	↑	↑	↓	✗	↑	↓	↓	–
JUN	na	↓	na	↑	✗	↓	na	↓	↑	✗	↓	na	↓	✓

Table S2: MSC Control Centrality values of selected nodes

Node	TC	VC	DC
ACAN	4,62E-10	6,43E-21	1,01E-03
ALP	8,19E-03	1,06E-18	7,10E-03
CEBPs	1,34E-07	3,46E-20	1,68E-07
CTNNB1	1,22E-02	2,65E-14	1,32E-02
PPARG	1,00E-03	2,73E-12	1,00E-02
RUNX2	1,29E-02	8,82E-04	7,94E-03
SOX9	8,06E-03	1,86E-03	1,55E-02
TP53	9,51E-02	2,64E-02	7,06E-02

Node	TC	VC	DC
ACAN	0.00143	0.00000	0.00135
ALP	0.00828	0.00000	0.00820
CEBPs	0.00037	0.00002	0.00019
CTNNB1	0.01340	0.00003	0.01294
PPARG	0.00116	0.00017	0.00066
RUNX2	0.01077	0.00016	0.01103
SOX9	0.01477	0.00233	0.01197
TP53	0.08131	0.01260	0.07733

TC,VC: 18000 iterations

DC: 2000 iterations with 50 minimum, dt: 0.1

Part II --Analyzing checks and balance mechanisms in plant signaling networks

The check and balance mechanisms in plants including the model plant Arabidopsis relies on the function of only few central network regulatory components that can be extracted out of the overall network topology. We added values for each of those basic nodes to show their individual influence to steer the networks behavior: TC, DC and VC. We show that this is now quantified and we give the values in the picture (from top to bottom at each node: TC; VC; DC).

In an experimental setting we can study how the plant copes with just *Pst* DC3000. However, under real world conditions other pathogens include other bacteria, fungi as well as virus infections. Furthermore, the plant has to grow well (central hormone auxin, mitigating immune defense). The effort against all these different stresses, pathogens and tasks has to be optimized, this has been achieved by evolution to yield the above simplified network and checks and balances for each pathway as revealed and quantified here by Jimena. Clearly, for instance, SA response is weak in the network.

Though this is good for survival in the wild, for crop yield it can be easily seen that directly spraying cytokinins when there is infection is beneficial to mitigate infection and fight *Pst* DC3000 [17]. This is hence a good strategy to protect crops against infection, in particular against gram negative bacteria such as *Pseudomonas syringae*. Jimena is also very helpful to analyze the involved hormonal protein-protein interaction networks [24]. Checks and balances optimize the plant response depending whether there is a gram negative pathogen attacking (cytokinin protects), a fungal pathogen or for instance draught stress (salicylic acid modulation is important). Moreover, Jimena allows to examine the centrality values for different nodes in the network according to the exerted control on the network: direct control (value centrality, VC), control via dynamics and network nodes (DC) or a combined control (TC; details on control types in mathematical appendix). For instance, the marker node PR1 shows high centrality values and some other nodes are pointed out by this analysis (data in Naseem et al., 2012; 2017).

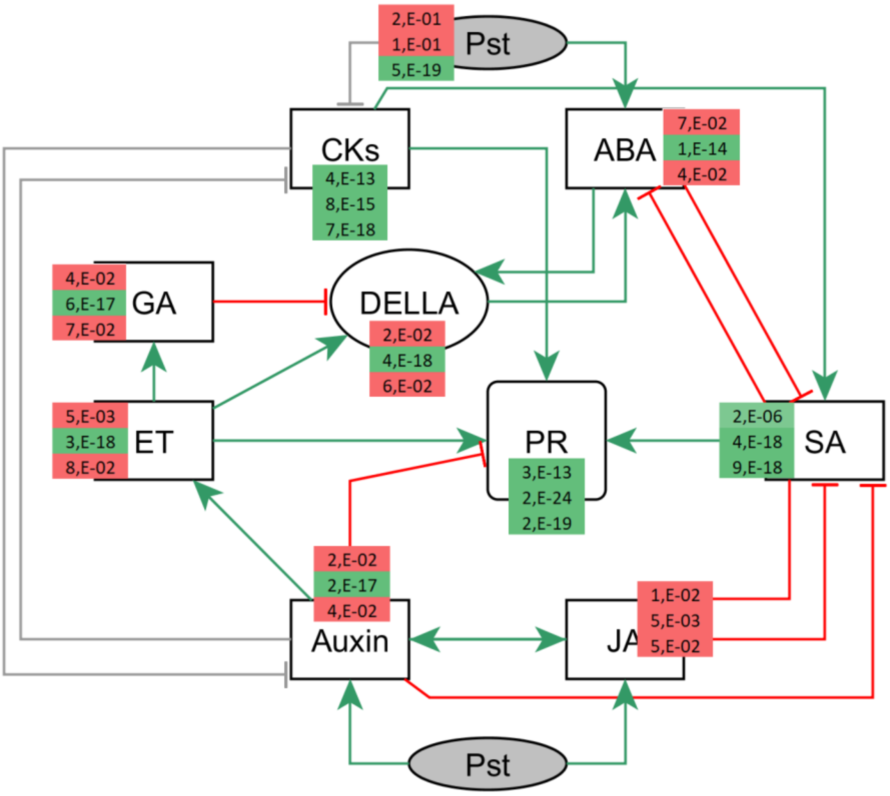

Supplementary Figure S2: Checks and balance analysis in a plant signaling network. Simplified overview network describing the general processes in *A.thaliana* during *Pst* DC3000 infection. Details see text.

Table S3: *Arabidopsis thaliana* Control Centrality Values of important hub nodes

Node	TC	VC	DC
**ABA**	0.04854	0.00058	0.04823
**Aux/IAA**	0.00105	0.00024	0.00029
**Auxins**	0.01807	0.00057	0.01833
**Ethylene**	0.05466	0.00021	0.05240
**Gibberelic Acid**	0.06983	0.00022	0.07102
**JA**	0.05565	0.00079	0.05382
**MYC2**	0.02489	0.00105	0.02402
**NPR1**	0.01889	0.00108	0.01697
**PR1**	0.00000	0.00000	0.00000
***Pst* DC3000**	0.00129	0.00092	0.00005
**SA**	0.00252	0.00141	0.00110
**TGA TF**	0.05002	0.00079	0.04694

TC, VC: 18,000 iterations; DC 2,000 iterations with 50 for the minimum in the formula.
The software Jimena2 was set such that the integral for TC and VC was approximated with 18000 iterations and for DC this integral was approximated with 2000 iteration where the minimum in this integral was approximated with 50 iterations. The step size (dt) is set to 1 for this test.

For the different type of centralities different integrals are to solve [25]. In order to solve them properly, a sufficient number of iterations is necessary in the Jimena Software package. If we start the calculations for the node centralities with increasing iteration numbers, we observe convergence of the values of the centrality for each node after two different runs with the same iteration number only if the iteration number is sufficiently large. This means for a practicable use of the Jimena software with respect to centrality calculations we increase the number of iterations and run the calculations twice. If the corresponding values is equal up to that digit that the calculation is supposed to be reliable on we have found a reasonable number of iterations that result in a sufficient accuracy of the values of the centralities of the nodes.

Part III --Analyzing immune response network control in murine dendritic cells

Control centrality analysis with Jimena reveals (Fig. S5A), that only the three major network stimuli (the immune response creating pathogen, fungus *Aspergillus fumigatus germinal tubes*, AFgt; stimulation by platelets from blood during sepsis by the pathogen; and tumor necrosis factor, TNF) among the components of interest show a combined high controlling influence (VC>10^{-3}) on the network topology and primary signal transduction function (DC>10^{-3}, see figure 5A). For optimal survival DC immune response should be rapid and dominated by the key external stimuli and this led to a selection for both high VC (direct control) and DC (dynamic control) and a strong combined TC (total control). Those three direct external stimuli (AFgt, platelets and TNF) furthermore express a strong involvement in the central signaling cascades (DC>10^{-3}) and are vital to a proper function of the signaling processes (TC>10^{-3}).

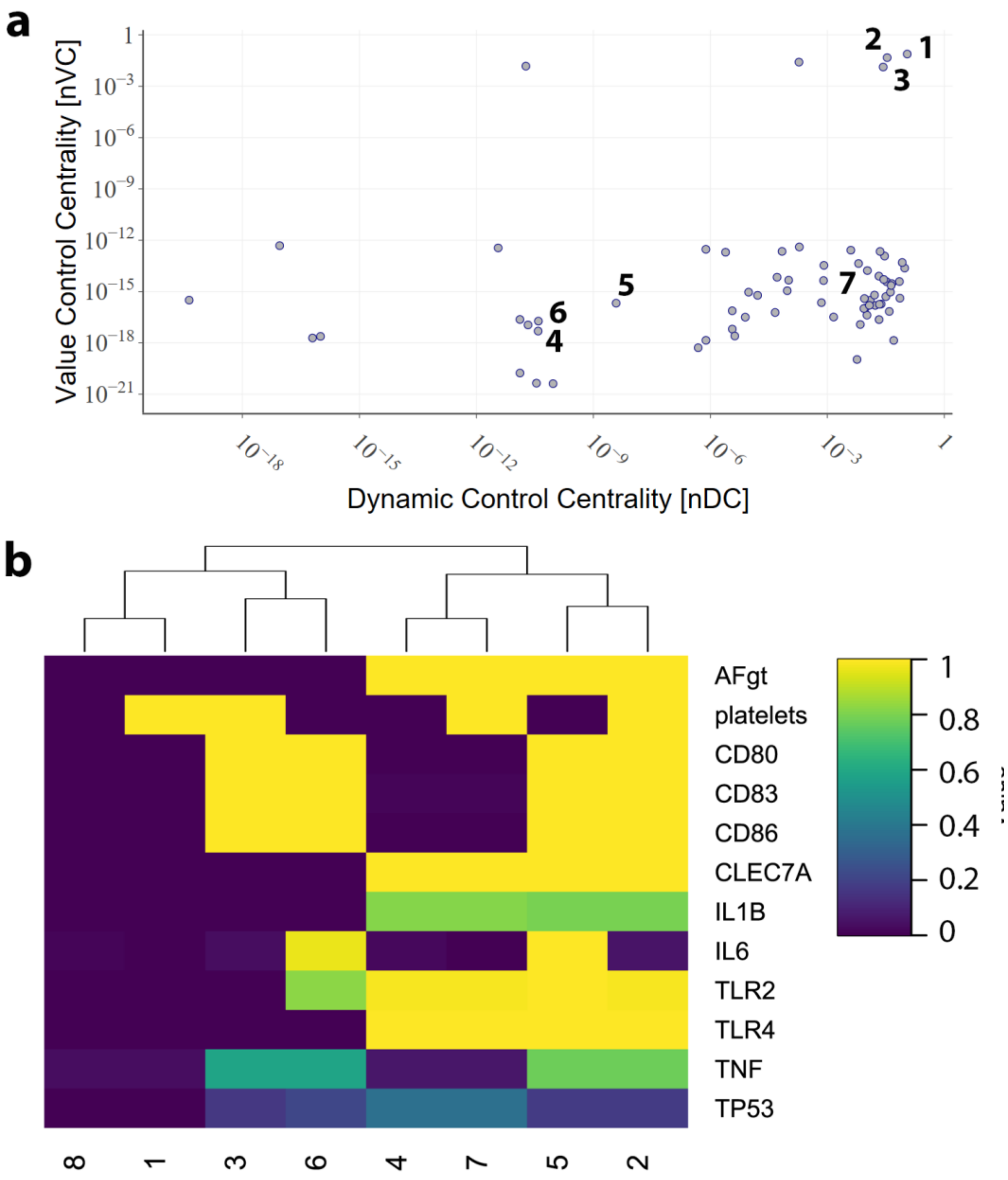

Supplementary Figure S3: Analyzing immune response network control in murine dendritic cells. Control Centrality and Stable Steady States in dendritic cells under infectious aspergillosis by Jimena helps to analyze and model how dendritic cells sense and discriminate *A. fumigatus* growth types. Analysis of key network nodes and their type of control in the dendritic cell signaling network: (A) Scatter plot illustrating Value Control Centrality (VC) against Dynamic Control Centrality (DC) values of the dendritic cell network. Highlighted data points are: 1- *A.fumigatus* germ tubes; 2. Platelets; 3. TNF; 4. CD80; 5. CD83; 6. CD86; 7. TP53. While 1-3 show very strong control values and 7 a major function in signal transduction pathways, components 4-6 have no prominent influence on the network. (B) Heatmap illustrating the SSSs of the network influenced by the two stimuli nodes A. fumigatus (Afgt) and Platelets. Activity values are color-coded with blue fields being completely inactive (value: 0.0) over green (value ~0.5) and yellow being fully active (value: 1.0). The primary nodes of interest (CD80, CD83, CD86, TLR2, TLR4 and IL6 show activity in four of ten states (SSS 5, 6, 7 and 10) with differences present at Afgt and Platelet Activity. SSS 1-3 present complete inactivity of all examined nodes. SSS 4 shows activation only in one component: Platelets. SSS 8 and 9 illustrate steady states with inactive maturation markers CD80, CD83 and CD86 as well as IL6 and TNF while Afgt, Dectin-1 IL1B, TLR2 and TLR4 are constantly upregulated.

The analysis of steady states lists 8 different steady states (Fig. S5B) which, when reduced only on the network key nodes (according biological significance), can be further condensed to only four general equilibria with pairs only differing regarding presence or lack of additional platelet activation of the DC immune response and those nodes that directly depend on their activity.

Table S4: Dendritic cell control. Centrality of important hub nodes and marker proteins

Node	VC	TC	DC
AF Germ Tubes	1,02E-01	1,27E-01	1,09E-01
CCL2	3,32E-21	0,00E+00	0,00E+00
CCL5	1,10E-16	8,12E-03	6,59E-03
CD80	6,10E-16	2,38E-11	3,37E-11
CD83	5,40E-19	1,15E-09	2,41E-10
CD86	2,05E-16	1,25E-11	1,05E-11
CXCL10	1,33E-18	0,00E+00	0,00E+00
Dectin-1	5,97E-13	9,08E-02	9,17E-02
IL1	6,51E-13	2,38E-02	5,23E-04
IL6	2,41E-15	1,12E-02	7,92E-03
Platelets	4,61E-02	8,75E-02	2,28E-02
TLR2	1,93E-14	4,34E-02	3,47E-02
TLR4	1,12E-16	3,46E-02	3,07E-02
TLR6	1,13E-19	6,84E-06	3,57E-06
TNF	1,32E-02	3,45E-02	2,49E-02
TP53	1,58E-15	3,06E-04	4,26E-04

Node	TC	VC	DC
AF Germ Tubes	0.10634	0.03792	0.10271
CCL2	0.00000	0.00000	0.00000
CCL5	0.00773	0.00000	0.00790
CD80	0.00007	0.00020	0.00000
CD83	0.00016	0.00010	0.00000
CD86	0.00009	0.00018	0.00000
CXCL10	0.00000	0.00000	0.00000
IL1	0.01867	0.00743	0.00480
IL6	0.02253	0.00011	0.02341
Platelets	0.11910	0.05860	0.01567
TLR2	0.02217	0.00091	0.02240
TLR4	0.00347	0.00014	0.00334
TLR6	0.00001	0.00000	0.00001
TNF	0.05484	0.00896	0.03082
TP53	0.00206	0.00033	0.00157

DC, VC, TC iteration conditions are as in Table S1. The step size (dt) is set to 1 in Jimena for this test.

Table S5: Steady state analysis of maturation markers

Group	1		2	3	4	
CCL5	0.000		1.367	0.0E+00	0.930	*
CD80	0.999	*	1.451	0.0E+00	0.999	*
CD83	0.995	*	0.900	0.0E+00	0.995	*
CD86	0.999	*	0.871	0.0E+00	0.999	
Dectin-1	0.412	*	-0.214	5.1E-01	0.412	*

There exist different groups of steady states regarding maturation markers such as CD80, CD83 and CD86 while in the other groups these markers are less active. *The step size (dt) is set to 0.2 instead of default value (0.1) for this test.

Supplementary Figure S4: T-cell Models examined by Jimena

Another application of Jimena regarding immune cells is that it can of course also simulate T-cell maturation (see also Karl and Dandekar, 2015). Such models can then be used to study for instance pharmacological intervention points. The Figure shown directly below just for illustration of Jimena´s modelling powers is the circuit network implemented by and reproduced from “Breitenbach et al., 2019; Analyzing pharmacological intervention points: A method to calculate external stimuli to switch between steady states in regulatory networks https://pubmed.ncbi.nlm.nih.gov/31310618/ as this was published with a creative commons licence).

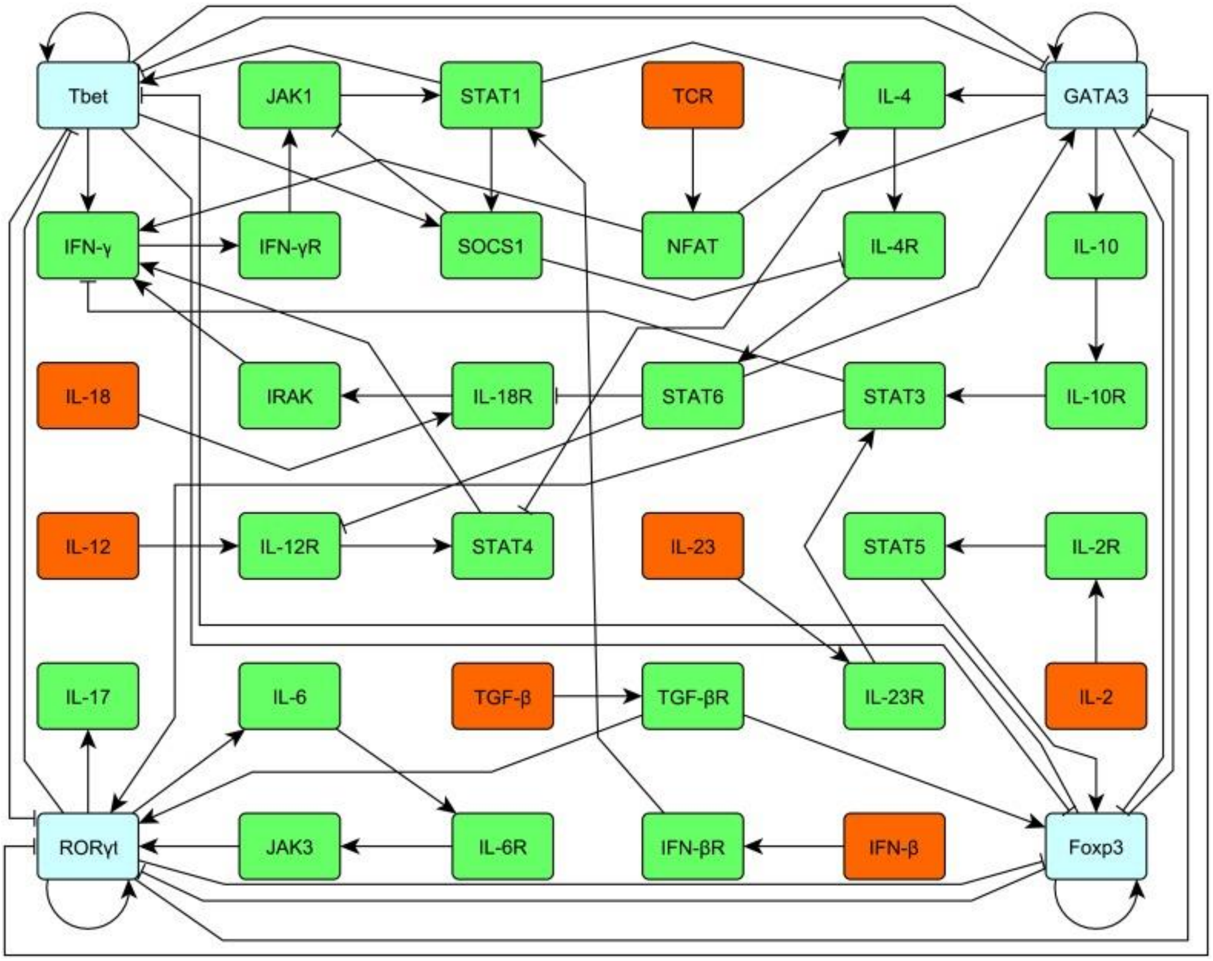

Mathematical Appendix

This appendix treats the above concepts (system states; centrality measures and dynamic simulations in general) from a mathematical point of view and assumes that the reader has familiarized himself with the introduction above and saw the biological examples reported in the paper and the online methods to have the background for the appendix.

Remarks on points of rest of ordinary differential equations, oscillations in networks and error bars of numerically located point of rests

According to Mendoza and Xenarios (2006) [26], we consider a system of ordinary differential equations

$$\begin{aligned} \frac{d}{\mathrm{dt}}x &= f(x) \\ x(0) &= x_0 \end{aligned} \qquad (1)$$

which models a network of nodes interacting with each other where $x(t): R \to R^n$ is a vector whose components are the activation level of the corresponding node $x_0 \in R^n$ and $f: R^n \to R^n$ is the continuously differentiable right-hand side of the differential equation with n a natural number as the dimension or number of nodes, respectively. In our simulations where the activation level is modeled by the state x, we observe that the activation levels converge to an equilibrium, that means that the activation levels do not change any more after a certain time and keep their values for all times left. We say that the system has gone to a position of rest. This is characterized by

$$0 = \frac{d}{\mathrm{dt}}x = f(x).$$

That means that the positions of rest are roots of the right hand-side f. Another interpretation is that the time derivative $\frac{d}{\mathrm{dt}}x$ is zero for all times. That means that the function x has to be constant for all times.

We distinguish three different types of positions of rest. There are instable ones. That means that a little deflection (within a small epsilon-interval) from the position of rest can cause that the system never reaches that position of rest again. There are stable ones. That means that a little deflection from the position of rest lets the system remain in a certain neighborhood around the position of rest for all times. In this case, we observe that the values of the corresponding quantities oscillate around their position of rest. The third type is called asymptotically stable. That means that after a little deflection, the system converges to the position of rest again.

In order to investigate the type of a position of rest, we need to know the behavior of the system at least within a small environment around the position of rest. We label a position

of rest by $\bar{x} \in R^n$. Having the Taylor series of f around $\bar{x}$, there is a small environment $E(\bar{x})$ around $\bar{x}$ such that the Taylor series up to the first term is a good approximation for f, that means

$$f(\varkappa) \approx f(\bar{x}) + \mathrm{Df}(\bar{x})(\varkappa - \bar{x})$$

where $\mathrm{Df}(\bar{x})$ is the Jacobian of f evaluated at $\bar{x}$ and $\varkappa \in E(\bar{x})$. As $\bar{x}$ is a point of rest and thus $f(\bar{x})=0$, we have that in a small environment around $\bar{x}$, the system (1) behaves like a homogeneous system of liner differential equations which is given by

$$\frac{d}{\mathrm{dt}}\varkappa = \mathrm{Df}(\bar{x})(\varkappa - \bar{x}).$$

After a coordinate transformation where we set $x = \varkappa - \bar{x},$, we have approximately

$$\frac{d}{\mathrm{dt}}(x + \bar{x}) = \mathrm{Df}(\bar{x})x$$

which is equivalent to

$$\frac{d}{\mathrm{dt}}x = \mathrm{Df}(\bar{x})x$$

as $\bar{x}$ is a constant. The general solution to a homogeneous system can always be calculated and the asymptotic behavior is characterized by the real part of the eigenvalues, roughly spoken. See textbooks about ordinary differential equations like Sideris(2013), Teschl(2012) [14,15] for more details about solving systems of homogeneous differential equations and their asymptotic behavior. The most important case for our purpose is that the eigenvalues of $\mathrm{Df}(\bar{x})$ are of the structure $a + ib$ where the real part $a<0$ and the imaginary part $b \in \Re$. Illustratively stated, the real part is a measure for the damping that means how fast the system reaches its point of rest again once deflected where $a=0$ means that there is no damping and the absolute value of the imaginary part $|b|$ is a measure for how fast the values x oscillate where $b=0$ means that there is no oscillation. The sign of b affects the direction of the oszillation's rotation. More detailed, the positions of rest are characterized by the eigenvalues $\lambda_k, k=1,2,...$, of the Jacobian $\mathrm{Df}(\bar{x})$ evaluated at the corresponding position of rest $\bar{x}$. We have an instable one, if the real part of one eigenvalue is greater than zero, i.e. $\mathrm{Re}(\lambda_k)>0$ for one k. We have an asymptotically stable one if all the real parts of the eigenvalues are less than zero, i.e. $\mathrm{Re}(\lambda_k)<0$ for all $k=1,2,...$. A stable position of rest is for purely imaginary eigenvalues, i.e. $\mathrm{Re}(\lambda_k)=0$ and $\mathrm{Im}(\lambda_k) \neq 0$ for all $k=1,2,...$ and if all the eigenvalues are pairwise different.

We illustrate the above discussion with two examples. We present a two dimensional system of ordinary differential equations, namely a Lotka-Volterra system, given by

$$\frac{d}{\mathrm{dt}}x_1 = \gamma x_1 - \beta x_1 x_2$$

$$\frac{d}{\mathrm{dt}}x_2 = \alpha x_1 x_2 - \delta x_2$$

where $\gamma, \beta, \alpha, \delta>0$. We define

$$f_1(x_1, x_2) := \gamma x_1 - \beta x_1 x_2$$

$$f_2(x_1, x_2) := \alpha x_1 x_2 - \delta x_2$$

and further

$$f(x_1, x_2) := \begin{pmatrix} f_1(x_1, x_2) \\ f_2(x_1, x_2) \end{pmatrix}.$$

In order to find the positions of rest, that means these points where the values of the quantities x_1, x_2 do not change any more for any time, we search for the roots of f, that means

$$f_1(x_1, x_2)=0$$

$$f_2(x_1, x_2)=0$$

and equivalently,

$$\gamma x_1 - \beta x_1 x_2=0$$

$$\alpha x_1 x_2 - \delta x_2=0$$

where we have two positions of rest. The first one is for $\bar{x}_1=0$ and $\bar{x}_2=0$. The second and interesting one is for $\bar{x}_1 = \frac{\delta}{\alpha}$ and $\bar{x}_2 = \frac{\gamma}{\beta}$.

For the mathematical characterization, we need the Jacobian of the system. The Jacobian is given by the first derivative as follows

$$\mathrm{Df}(x_1, x_2) = \begin{pmatrix} \frac{\partial}{\partial x_1} f_1(x_1, x_2) & \frac{\partial}{\partial x_2} f_1(x_1, x_2) \\ \frac{\partial}{\partial x_1} f_2(x_1, x_2) & \frac{\partial}{\partial x_2} f_2(x_1, x_2) \end{pmatrix}$$

where $\frac{\partial}{\partial x_1}$ is the partial derivative with respect to x_1 and $\frac{\partial}{\partial x_2}$ is the partial derivate with respect to x_2. For the Lotka-Volterra system, we obtain

$$\mathrm{Df}(x_1, x_2) = \begin{pmatrix} \gamma - \beta x_2 & -\beta x_1 \\ \alpha x_2 & \alpha x_1 - \delta \end{pmatrix}.$$

Evaluating $\mathrm{Df}(x_1, x_2)$ at the point of rest $(x_1, x_2) = \left(\frac{\delta}{\alpha}, \frac{\gamma}{\beta}\right)$ for our Lotka-Volterra system, we obtain

$$\mathrm{Df}\left(\frac{\delta}{\alpha}, \frac{\gamma}{\beta}\right) = \begin{pmatrix} 0 & -\frac{\beta\delta}{\alpha} \\ \frac{\alpha\gamma}{\beta} & 0 \end{pmatrix}.$$

The eigenvalues of $\mathrm{Df}\left(\frac{\delta}{\alpha}, \frac{\gamma}{\beta}\right)$ are $\lambda_1 = -\sqrt{-\gamma\delta} = -i\sqrt{\gamma\delta}$ and $\lambda_2 = \sqrt{-\gamma\delta} = i\sqrt{\gamma\delta}$ and therefore purely imaginary and pairwise different. That means that x_1 and x_2 oscillate around the point of rest $\left(\frac{\delta}{\alpha}, \frac{\gamma}{\beta}\right)$ once deflected at most up to certain distance from the point of rest $\left(\frac{\delta}{\alpha}, \frac{\gamma}{\beta}\right)$.

The next example is according to the dynamics described in [Mendoza and Xenarios(2006)]. We have

$$\frac{d}{dt}x_1 = \frac{-\exp\left(\frac{1}{2}h\right)+\exp\left(-h\left(1-\frac{1+\beta}{\beta}\frac{\beta x_2}{1+\beta x_2}-\frac{1}{2}\right)\right)}{\left(1-\exp\left(\frac{1}{2}h\right)\right)\left(1+\exp\left(-h\left(1-\frac{1+\beta}{\beta}\frac{\beta x_2}{1+\beta x_2}-\frac{1}{2}\right)\right)\right)} - x_1$$

$$\frac{d}{dt}x_2 = \frac{-\exp\left(\frac{1}{2}h\right)+\exp\left(-h\left(\frac{1+\alpha_{12}+\alpha_3}{\alpha_{12}+\alpha_3}\frac{\alpha_{12}x_1+\alpha_3 x_3}{1+\alpha_{12}x_1+\alpha_3 x_3}-\frac{1}{2}\right)\right)}{\left(1-\exp\left(\frac{1}{2}h\right)\right)\left(1+\exp\left(-h\left(\frac{1+\alpha_{12}+\alpha_3}{\alpha_{12}+\alpha_3}\frac{\alpha_{12}x_1+\alpha_3 x_3}{1+\alpha_{12}x_1+\alpha_3 x_3}-\frac{1}{2}\right)\right)\right)} - x_2 \tag{2}$$

$$\frac{d}{dt}x_3 = \frac{-\exp\left(\frac{1}{2}h\right)+\exp\left(-h\left(\frac{1+\alpha_4}{\alpha_4}\frac{\alpha_4 x_4}{1+\alpha_4 x_4}-\frac{1}{2}\right)\right)}{\left(1-\exp\left(\frac{1}{2}h\right)\right)\left(1+\exp\left(-h\left(\frac{1+\alpha_4}{\alpha_4}\frac{\alpha_4 x_4}{1+\alpha_4 x_4}-\frac{1}{2}\right)\right)\right)} - x_3$$

$$\frac{d}{dt}x_4 = \frac{-\exp\left(\frac{1}{2}h\right)+\exp\left(-h\left(\frac{1+\alpha_{14}}{\alpha_{14}}\frac{\alpha_{14}x_1}{1+\alpha_{14}x_1}-\frac{1}{2}\right)\right)}{\left(1-\exp\left(\frac{1}{2}h\right)\right)\left(1+\exp\left(-h\left(\frac{1+\alpha_{14}}{\alpha_{14}}\frac{\alpha_{14}x_1}{1+\alpha_{14}x_1}-\frac{1}{2}\right)\right)\right)} - x_4$$

where we set $\alpha_{12} = \alpha_4 = \alpha_{14}=1$, $\beta=10$ and $h=10$. In Figure A1, we have the schematic of the network.

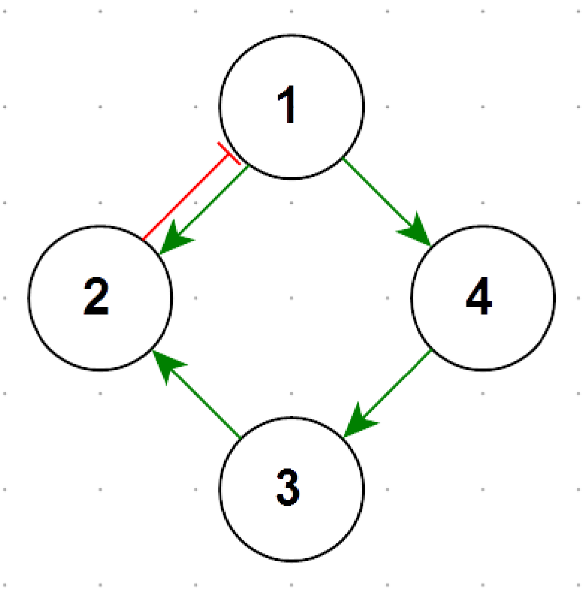

Figure A1: Schematic of the network

We use Wolfram Mathematica, namely FindRoot, to find numerically a root of the right hand-side of (2) which is rounded given by

$$(\bar{x}_1,\bar{x}_2,\bar{x}_3,\bar{x}_4) = (0.196559, 0.13822, 0.0756964, 0.147855). \tag{3}$$

By Wolfram Mathematica, we calculate the Jacobian of the right hand-side of (2), evaluate it at $(\bar{x}_1,\bar{x}_2,\bar{x}_3,\bar{x}_4)$ and obtain for its eigenvalues the following rounded values $(\lambda_1,\lambda_2,\lambda_3,\lambda_4) = (-1.6891+1.5129i, -1.6891-1.5129i, -0.3109+1.5129i, -0.3109-1.5129i)$. Therefore, we have an asymptotically stable point of rest. In other words, we can say that starting our simulation close enough to the point of rest $(\bar{x}_1,\bar{x}_2,\bar{x}_3,\bar{x}_4)$, the state (x_1,x_2,x_3,x_4) performs a

damped oscillation around $(\bar{x}_1, \bar{x}_2, \bar{x}_3, \bar{x}_4)$ converging to the values $(\bar{x}_1, \bar{x}_2, \bar{x}_3, \bar{x}_4)$ when time goes forward. We can see this in Figure A2 which is calculated with the Mathematica function NDSolve where

$$(x_1(100), x_2(100), x_3(100), x_4(100)) = (0.196559, 0.13822, 0.0756964, 0.147855)$$

and the initial point is $(x_1(0), x_2(0), x_3(0), x_4(0)) = (0,0,0,0)$.

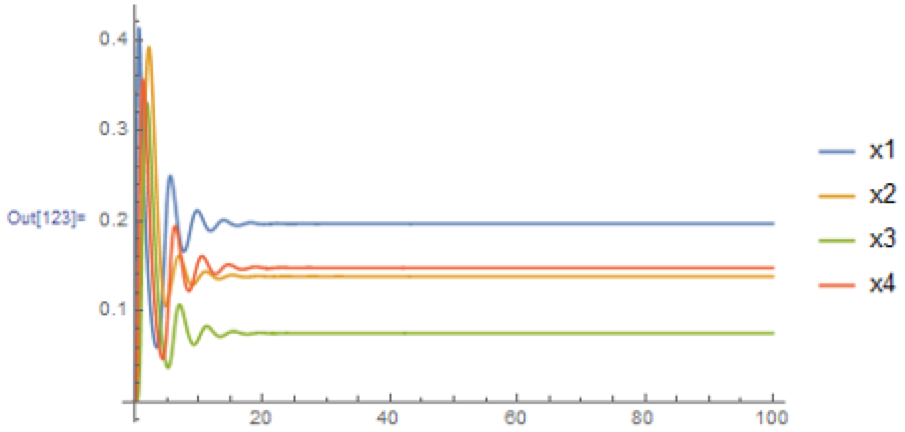

Figure A2 Plot of the solution to (2) calculated with NDSolve

If we do not use a solver to find the roots of the right hand-side f but a solver for simulating the network according to (1) and thus calculate the whole time-dependent course of x, we face some numerical instabilities using certain numerical methods for solving (1). That means that the numerical solution to a system of ordinary differential equation does not reproduce the asymptotic behavior of the analytic solution to the corresponding system. In fact, the correct asymptotic behavior is crucial to determine the points of rest. The central topic of this issue is the stiff (differential) equation. We illustrate the basic problem with an example. We consider the one dimensional system

$$\frac{d}{\mathrm{dt}} x = \lambda \left(x - \frac{1}{3}\right) \qquad x(0) = \frac{4}{3} \tag{4}$$

which is solved by $x(t) = \exp(\lambda t) + \frac{1}{3}$ whose asymptotic behavior is given by $\lim_{t\to\infty} x(t) = \frac{1}{3}$ if λ<0. In order to solve (4) numerically, we have to discretize its differential equation. There are several possibilities. We choose the following

$$\frac{x^{k+1} - x^k}{\Delta t} = \lambda \left(x^k - \frac{1}{3}\right) \tag{5}$$

where x^k is the approximated value of the analytical solution $x(k\Delta t)$ with $\Delta t \in R^+$ and $k \in N_0$. From (5), we can calculate the value x^{k+1} from the known previous time step where $x^0 = \frac{4}{3}$ as follows

$$x^{k+1} = \Delta t \lambda \left(x^k - \frac{1}{3}\right) + x^k$$

which is equivalent to

$$x^{k+1} = (1+\Delta t\lambda)x^k - \frac{1}{3}\Delta t\lambda. \quad (6)$$

Using mapping rule (6), we iteratively obtain

$$k - 1(1+\Delta t\lambda)^n + (1+\Delta t\lambda)^k x^0$$

$$= -\frac{1}{3}\Delta t\lambda \frac{1-(1+\Delta t\lambda)^k}{1-(1+\Delta t\lambda)} + (1+\Delta t\lambda)^k x^0$$

$$= -\frac{1}{3}\Delta t\lambda \frac{1-(1+\Delta t\lambda)^k}{-\Delta t\lambda} + (1+\Delta t\lambda)^k x^0$$

$$= \frac{1}{3} - \frac{1}{3}(1+\Delta t\lambda)^k + (1+\Delta t\lambda)^k x^0$$

where we used the Geometric series. If $|1+\Delta t\lambda|<1$, then $\lim_{k\to\infty} x^k = \frac{1}{3}$ and thus the numerical solution to (4) provides the correct asymptotic behavior. For example, if $\lambda = -4$, then for $\Delta t < \frac{1}{2}$ the scheme (6) for calculating the numerical solution to (4) provides the correct asymptotic behavior. The key point is that we have to choose the step size sufficiently small to obtain a numerical solution with a correct asymptotic behavior. This is illustrated in the following example.

Now, we can also see this stability effects for our system (2). We solve (2) for $t \in [0,100]$ and the initial point $\left(x_1(0), x_2(0), x_3(0), x_4(0)\right) = (0,0,0,0)$ with the Mathematica function NDSolve where we set the options StartingStepSize → Δt, Method → {"FixedStep", Method → "ExplicitEuler"}. In other words, we use an explicit Euler scheme with fixed step size which means that $x^{k+1} = x^k + \Delta t \cdot f\left(x^k\right)$. In Figure A3 to Figure A8, we can see the numerical solutions for different step sizes Δt. The convergence to the point of rest can be seen first if the step size Δt is sufficiently small. Figure A8 looks then identically to Figure A2.

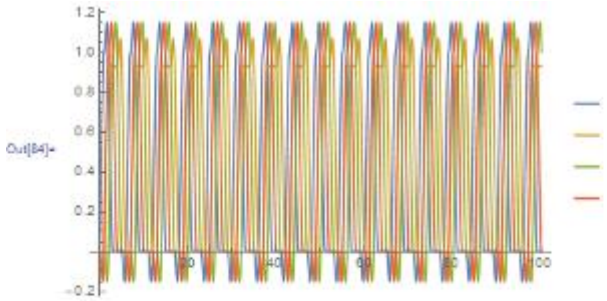

Figure A3 Plot of the solution to (2) for Δt = 1 with explicit Euler method

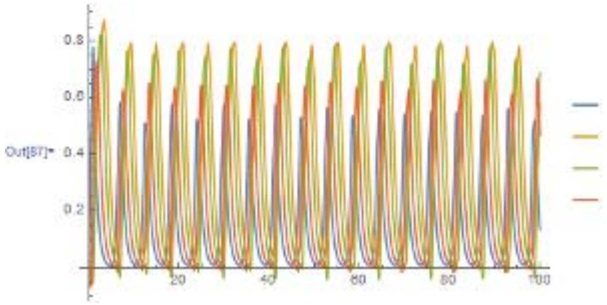

Figure A4 Plot of the solution to (2) for Δt = 0.5 with explicit Euler method

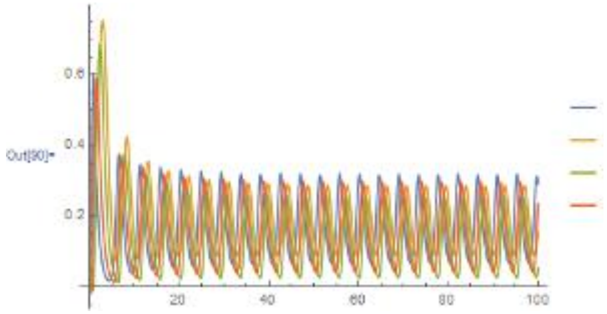

Figure A5 Plot of the solution to (2) for Δt = 0.3 with explicit Euler method

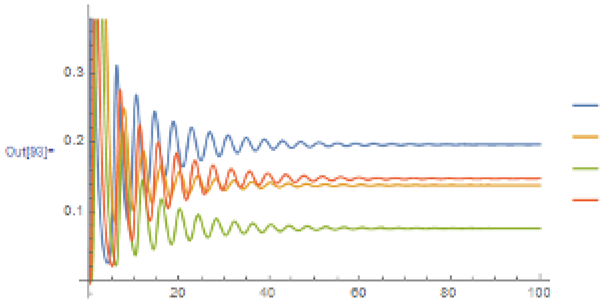

Figure A6 Plot of the solution to (2) for Δt = 0.2 with explicit Euler method

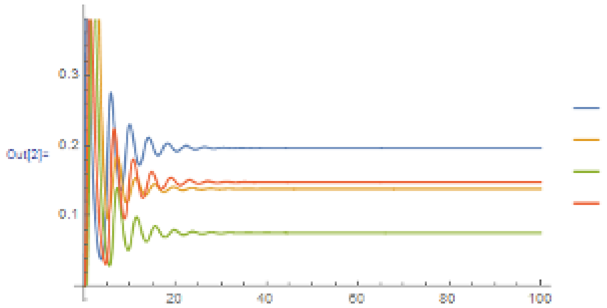

Figure A7 Plot of the solution to (2) for Δt = 0.1 with explicit Euler method

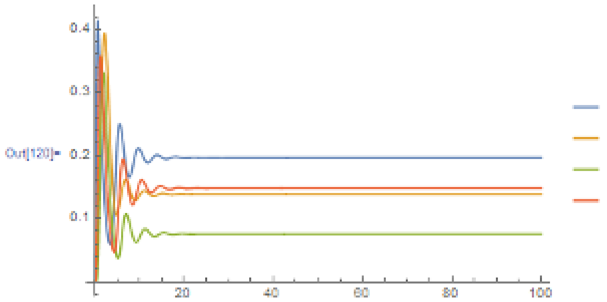

Figure A8 Plot of the solution to (2) for Δt = 0.001 with explicit Euler method

Plots of the numerical solution to (2) for different step sizes Δt

Having numerical results like Figure A3, Figure A4 or Figure A5 for any step size Δt and simulation time, that means undamped oscillations, especially then it is worth using an ODE-solver from Matlab like ode45 or NDSolve from Mathematica to solve the system of differential equations belonging to the given network in order to check the numerical results obtained from the tool used to analyze the given network.

Due to the fact that we approximate the time derivatives $\frac{d}{\mathrm{dt}}x(t)$ by their corresponding difference quotients $\frac{x(t+\Delta t)-x(t)}{\Delta t}$ for the numerical solution, we may not expect that the numerical solution equals exactly the analytical solution. As a consequence it is reasonable to

give the values of the nodes' activation level of the numerically located points of rest with some error bars. Then, from the numerical perspective, it is reasonable that we distinguish points of rest pairwise if and only if there is at least one component of the points of rest where the corresponding error bars do not overlap. Next, we present a procedure how to obtain an idea in what order of magnitude the error bars are. In Table A1, we have the values for $\left(x_1(100), x_2(100), x_3(100), x_4(100)\right)$, which serve as an estimation for the values of the point of rest. These values converge to the values for our point of rest calculated by the Mathematica function FindRoot, see (3). If we compare the values for Δt=0.2 with Δt=0.1, we realize that the difference is about $\pm 10^{-5}$. Therefore, if we perform the calculations for (2) with Δt=0.1 and with different initial points in order to find several points of rest, an error bar in the order of magnitude of $\pm 10^{-5}$ might be reasonable for the identified point of rest with its best values for Δt=0.1 in Table A1. To consider the error bars evaluation is reasonable. The solutions become mathematically similar, if the values are close to the machine precision. Currently this is 10^{-16} on common desktop computers. Numbers close to that value can be considered as zero. Steady states that do not differ in any component by the mentioned error bars can be considered as one class of the same steady state and any one of this class can be taken as a representative for further analysis.

Δt	$x_1(100)$	$x_2(100)$	$x_3(100)$	$x_4(100)$
0.21	0.19673	0.13827	0.0757159	0.148116
0.20	0.196604	0.138243	0.0757134	0.147945
0.10	0.196559	0.13822	0.0756964	0.147855

Table A1 Values of the nodes' activation level at the final time t=100

In general, it is a reasonable procedure to do the simulation for a network with the same initial point but with different step sizes Δt and compare the differences in the corresponding values of the nodes' activation level to get an idea for the order of magnitude of the error bars for the values of the corresponding nodes' activation level of the point of rest. Of course the step sizes for the calculation for the estimation of the error bar should not be too far from the step size with which the real simulation is performed such that the error bar becomes not too big or too small and the method is still stable.

If we take the absolute value of the difference between the corresponding components of two points of rest starting each form two different initial points from which the network converges to the particular point of rest and if this absolute value gets smaller and smaller when we perform the simulation for the corresponding given initial values of the network's nodes with smaller and smaller Δt, then the two different points of rest might be the same in fact as the difference comes from the numerical error. But if this absolute value converges to

a fixed number whose absolute value is greater than zero although decreasing Δt, then the points of rest might really be different ones.

The procedure described above is one possibility to estimate the numerical error. In order to compare the numerical results, that means the located points of rest, with data from an experiment, we have to be aware of the error bars with which the experimental data is provided. Normally, those error bars are from an order of magnitude of about 10% of the maximum activation level. That means in our case where the activation level is between 0 and 1, that the error bar is about ± 0.1 for the experimental data. Thus, in our example, we can neglect our numerical error of about $\pm 10^{-5}$ compared to ± 0.1. In general, if we compare numerical results to experimental data, it is reasonable to compare numerical and experimental error bars and provide the numerical data with the rounded common error (which here would neglect the small first error of 10^{-5}).

Dealing with different networks whose dynamics are according to Mendoza and Xenarios(2006) [26], we have not obtained any undamped oscillations and thus we assume that they are excluded within this model proposed in Mendoza and Xenarios(2006) [26]. This is plausible because each node is equipped with a decay.

To drive the network from one point of rest to another, we have to introduce some external controls which affect the activity level of a certain set of nodes in such a way that the whole network is driven from one point of rest to another. In other words, the perturbation of this certain set of nodes has to be strong enough such that the point of rest, in which the network is, is left and is brought close enough to the desired point of rest such that the network gets into that one. This is one possible way to switch between points of rest of a network and a corresponding systematical framework is discussed in Breitenbach et al. (2019) [27].

We stress that once the network is in a point of rest, it cannot switch its current state by itself based on our assumptions. That means that without external stimuli the system cannot leave the current steady state via construction of the model. This can be seen for example as follows. If the network is in a point of rest $\bar{x}$, then we have that the right hand-side $f(\bar{x})=0$. Thus, by (1), we have that the state x is constant, namely $x(t) \equiv \bar{x}$ for all the times left. The reason for this is that as the derivative of the constant $\bar{x}$ with respect to time equals zero as well as the right-hand side of (1) since it is a root of the right-hand side and thus the constant is a solution of (1) for the rest of the time once the values of the state x has reached $\bar{x}$. In particular, that means that there cannot occur oscillations between points of rest without external stimuli.

This analysis is for the interested reader. However, of course the problem of instability of numerical methods due to the step size parameter is well known and documented. For example in Kraaijevanger et. al (1987) [28].

Comparison of Boolean networks with continuous models such as SQUAD simulations

In this section, we discuss how to compare Boolean networks with the continuous networks with a dynamic governed by differential equations like SQUAD. Moreover, the same interaction graph is once translated into a Boolean network where the dynamic is given by Boolean functions and once in a continuous model where the governing dynamic consists of ordinary differential equations. We discuss how to find a corresponding continuous equivalent for OR and AND connections which are used in Boolean networks. The need for this discussion comes from the fact that, roughly spoken, AND connections are some kind of discontinuous operation and are straightforward to implement in Boolean networks but not for continuous models where the straight forward motive is rather the OR connection.

In the following we discuss the details.

If two nodes correspond to an OR connection, then the corresponding output is always an active child node except both parent nodes are inactive. An example from biology, where such a motive is useful is the expression of a gene that is downstream of different signaling pathways and the activity of each single pathway is sufficient for the expression of the corresponding gene. The corresponding interaction graph can be seen in **Error! Reference source not found.**. The simulation with Jimena where we introduce a permanent activation of strength 1 to Input 1 or Input 2 gives the desired output profile of the node Output as described above. In Boolean network dynamics the same behavior is given if two input nodes are connected by an OR function.

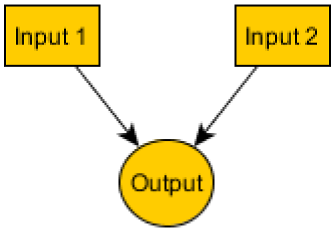

Figure A9 Schematic of an OR connection of two input nodes

If two nodes are connected with an AND connection, we would like to model the fact that for activation of the child node the activation of both parent nodes is needed. A node is active if its activity level, which is usually between 0 and 1, is above 0.5 and is inactive if its activity level is below 0.5. A biological example for an AND motive is that a product of a process can only be there if there is the enzyme and the corresponding substrate present. A second example is that two proteins need to be

present to form a heterodimer that is then biologically active. An advantage is that in the continuous formulation, we have the possibility to interpolate sharply between zero and one. This means we can adapt the non-linearity of the switch behavior for the case where some threshold value or a concentration of a product sharply increases. The corresponding schematic is given in Figure A10. In order to obtain the desired output behavior of the network, which means that the node Output is only active if the two nodes Input 1 and Input 2 are active, we use two extra technical nodes. The first one is the node System node which is always constantly fully activated and is used to activate the node Auxiliary node. Now as long as the Auxiliary node is active the node Output cannot be active. However, if Input 1 is active the Auxiliary node is inhibited and then the node Output node can be activated by the node Input 2 if this node is active. If we have a permanent activation of System node, a simulation with Jimena shows our desired output behavior.

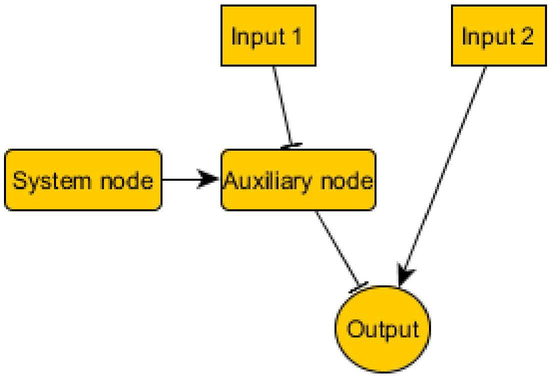

Figure A10 Schematic of an AND connection of two input nodes

Next, we discuss how to design OR and AND connections if there are more than two input nodes for continuous network dynamics. While an OR connection for more than two input nodes is straightforward, which means that all the Input nodes are connected with an activating connection with the output node, the AND connection for more than two input nodes works as follows. The first two input nodes are connected by an AND connection analogous to Figure A10. Then the output of this network is used as an input for a second AND connection where for the second input node the third node of our nodes that are supposed to be connected via an AND connection is taken. This procedure is repeated until all the nodes are connected. An example for three nodes can be seen in Figure A11 where we have an auxiliary node for each AND connection (numbered accordingly) with the same functionality as in the case depicted

in Figure A10 and still one System node constantly activated that activates all the Auxiliary nodes. A simulation with Jimena where the System node has a constant activity level of 1 gives the desired output, which means that the activity level of the node Output is active as long as the activity level of any input node is below 0.5.

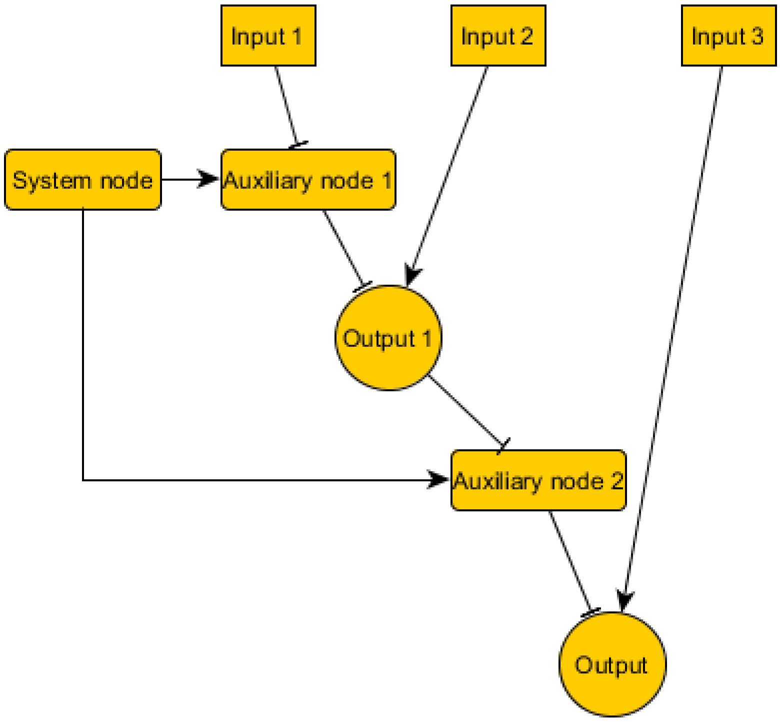

Figure A11 Schematic of an AND connection of three input nodes

We remark that an AND connection can be also realized with the connection depicted in Figure A12. For the simulation System node is always constantly activated with an activity level equal 1. Here the input nodes are connected more symmetrically than in the graph shown in Figure A10.However in our experience the output reacted less sensitive with respect to the input activity which means that for input activity around 0.5 the output of the graph from Figure A10 had a better switch behavior that we would like to have in order to obtain a behavior that corresponds to a logical AND connection. For example, if we choose 0.3 for the activity level of Input 1 and 1 for the activity level of Input 2, then the activity level of Output of the connection of Figure A12 is about 0.33 and of Output of the connection in Figure A10 is 0.06. We obtain a very strong switching behavior of the AND connection depicted in Figure A10 if the input node that usually has a strong activity level, which means almost 1, is Input 2, for example constitutively expressed proteins, and the input node that has a moderate activity level, which means around 0.5, is Input 1.

Next, we remark that the AND connection of the kind in Figure A12 is that each input node has its auxiliary node connected with an inhibiting t-shaped arrow and all the auxiliary nodes are connected with an inhibiting t-shaped arrow with the output node. One system node that has a constant activity level of 1 activates all the auxiliary nodes.

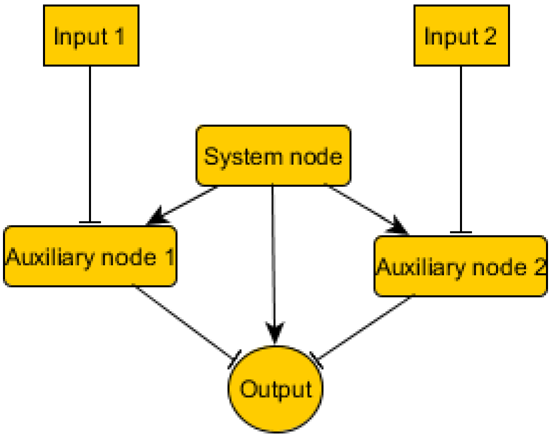

Figure A12 Schematic of another AND connection

The sensitivity of the output node can be set with the parameter “SQUAD steepness” in Jimena or equivalently on the equation level with the parameter h in Equation 3 of [Mendoza L, Xenarios I. A method for the generation of standardized qualitative dynamical systems of regulatory networks] in the equation that models the activity level for Output. The bigger the value for the SQUAD steepness or h is, the more the output behavior corresponds to a switch. This means that small inputs for the output node are mapped to an activity level of Output almost equal zero and moderate input activity which is only a little bit above 0.5 is amplified to an activity level of Output of almost 1. The closer SQUAD steepness or h, resp., is to 1, the input-output-amplification behaves almost linearly. That means that small input activity levels correspond to a small activity level of Output, moderate input activity to moderate output activity and only strong input activity results in a strong activity level of Output.

For completeness we discuss how to design an NOR and a NAND connection. This means we would like to have the opposite output of an OR connection or an AND connection, respectively. We start with the NOR connection depicted in Figure A13. The result of the OR connection that is available in the node “Inter_node” is negated in the node Output. This works as follows. The System node, which has always a constant activity level of 1 activates the node Output. However, if the result of the OR connection

of the two input nodes is an active Internode, this node inhibits the Output which is associated with a negating of the OR connection.

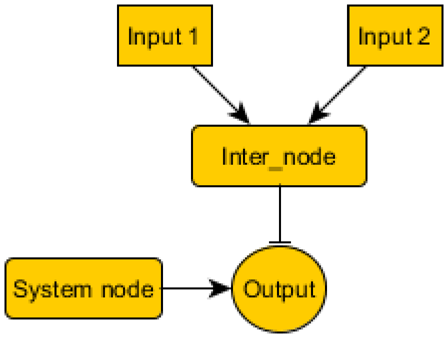

Figure A13 Schematic of a NOR connection of two input nodes

The NAND connection is designed from an AND connection with the same idea and depicted in Figure A14.

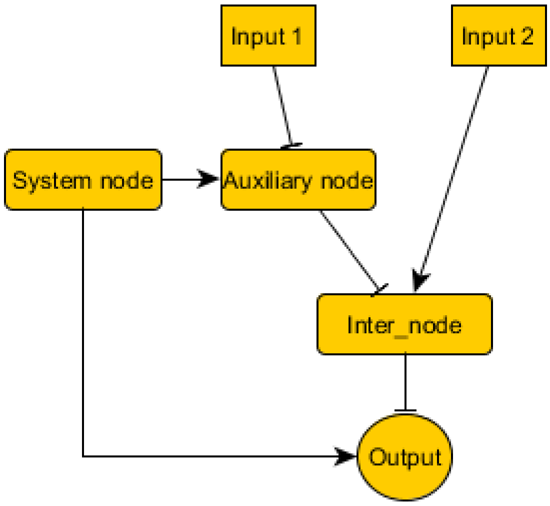

Figure A14 Schematic of a NAND connection of two input nodes

Finally, we would like to demonstrate with a small example that also the steady states analysis is affected by the kind of node connection. That especially means that the number of steady states can differ for OR and AND connections. We demonstrate the importance in the modelling process to choose the suitable connections of two nodes that corresponds best to the considered biological model if continuous model dynamics is used. That means we have to think about if the child node is supposed to correspond to an AND or rather OR behavior in order to obtain reasonable steady states that correspond to the biological system modeled with the corresponding network.

We start with an example where the number of steady states differs. The network where the input for the network is associated with an OR connection is

depicted in Figure A15 and the network where the input is associated with an AND connection is depicted in Figure A16. We just consider the values of the nodes Node A and Output since the others are technical nodes. For the simulation with Jimena of the AND case we again let the System node have a constant activity level given by 1. The OR case provides two steady states where in the first one Output and Node A both have the activity level 1 (rounded) and in the second case both 0 (rounded). The AND case just provides one steady state where the node Output and Node A have the value 0.

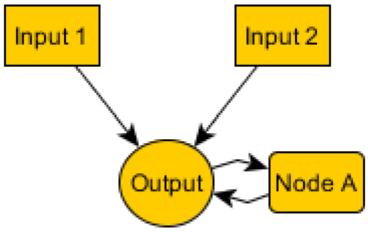

Figure A15 Input with an OR connection

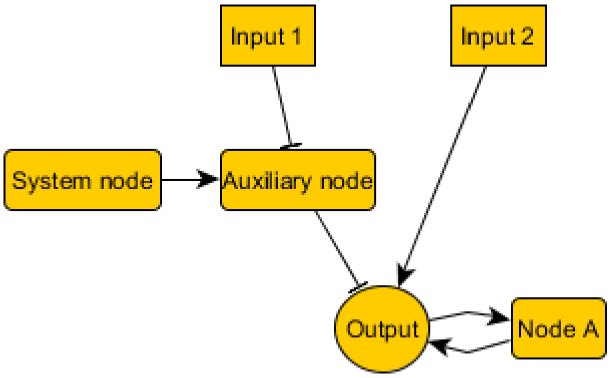

Figure A16 Input with an AND connection

In our second example we have the same number of steady states but the active nodes are different. The basic structure is shown in Figure A17 and the detailed connection is shown in Figure A18 (OR case) and in Figure A19 (AND case). In the following we use rounded values and we just consider the values of the nodes Node A, Node B and Output since the other are technical nodes. In the simulation with Jimena the System node is set to a constant activity level given by 1. Both networks, depicted in Figure A18 and Figure A19 have three steady states, where in one steady state the values of Node A, Node B and Output are all 0 and in another one all are 1. In the third steady state the difference is, that in the AND case Node A has value 1 and the other two have value 0 and in the OR case Node A has value 0 and the other two have value 1.

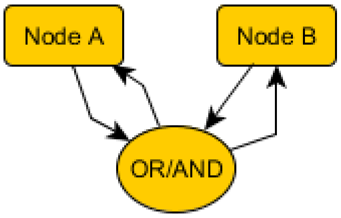

Figure A17 Basic OR/AND schematic for the second steady state example

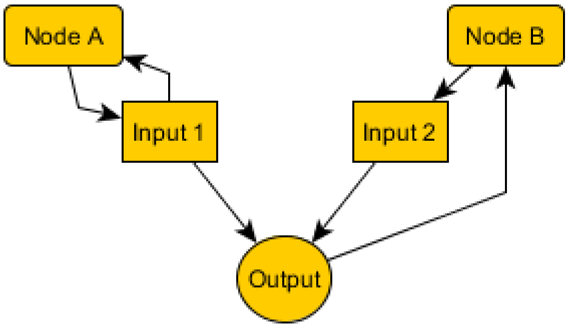

Figure A18 OR connection for the second steady state example

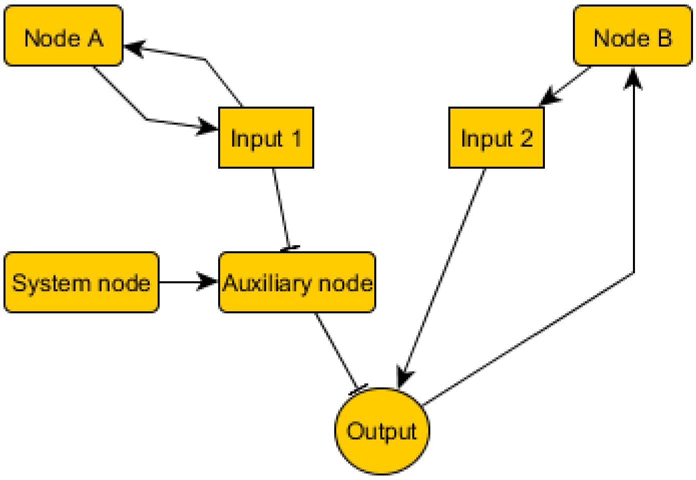

Figure A19 AND connection for the second steady state example

Further analysis

We implemented the methodology proposed by Mendoza and Xenarios (2006) [26] in order to show that with a minor adjustment this method is perfectly capable of reconstructing an AND gate in a continuous frame. This is to show a different perspective on how the logical gates can be obtained in quantitative simulations. The clean behavior of Boolean variables is attainable with the continuous quantitative network but it is necessary that the user carefully curate its model in order to correctly interpret the relations between nodes.

The equation used to transform a Boolean network into a continuous system is the following:

$$\frac{dx_i}{dt} = \frac{-e^{0.5h} + e^{-h(\omega_i - 0.5)}}{(1 - e^{0.5h})(1 + e^{-h(\omega_i - 0.5)})} - \gamma_i x_i$$

$$\omega_i = \begin{cases} \left(\frac{1+\sum \alpha_n}{\sum \alpha_n}\right)\left(\frac{\sum \alpha_n x_n^a}{1+\sum \alpha_n x_n^a}\right)\left(1 - \left(\frac{1+\sum \beta_m}{\sum \beta_m}\right)\left(\frac{\sum \beta_m x_m^i}{1+\sum \beta_m x_m^i}\right)\right) & \S \\ \left(\frac{1+\sum \alpha_n}{\sum \alpha_n}\right)\left(\frac{\sum \alpha_n x_n^a}{1+\sum \alpha_n x_n^a}\right) & \S\S \\ \left(1 - \left(\frac{1+\sum \beta_m}{\sum \beta_m}\right)\left(\frac{\sum \beta_m x_m^i}{1+\sum \beta_m x_m^i}\right)\right) & \S\S\S \end{cases}$$

$$0 \le x_i \le 1$$

$$0 \le \omega_i \le 1$$

$$h, \alpha_n, \beta_m, \gamma_i > 0$$

$\{x_n^a\}$ is the set of activators of x_i

$\{x_n^i\}$ is the set of inhibitors of x_i

§ is used if x_i has activators and inhibitors

§§ is used if x_i has only activators

§§§ is used if x_i has only inhibitors

As it is biologically more accurate this method is designed to assume that activating partners are related in an OR form. This means that one of the parents can activate the child node and there is no need for all of them to be active at the same time. This can be seen in this part of the equation for the weight ω_i:

$$\left(\frac{1+\sum \alpha_n}{\sum \alpha_n}\right)\left(\frac{\sum \alpha_n x_n^a}{1+\sum \alpha_n x_n^a}\right)$$

It is nevertheless possible to recreate the AND behavior. One just has to create a product of the factors that form the AND gate. So using the previous equation one would just need to also consider the products of activation terms. For example in the case of two factors one would have the following equation for the weight ω_i:

$$\left(\frac{1+\sum\alpha_n}{\sum\alpha_n}\right)\left(\frac{\sum\alpha_n x_n^a}{1+\sum\alpha_n x_n^a}\right) * \left(\frac{1+\sum\alpha_n}{\sum\alpha_n}\right)\left(\frac{\sum\alpha_n x_n^a}{1+\sum\alpha_n x_n^a}\right)$$

We implemented this change in R and plot the result. We can see that the AND behavior is correctly captured by the equation. It is basically a continuous AND gate, I also send a paper where they do a similar analysis using Hill functions.

Now, one might argue that changing the code of the program is difficult so including the extra system nodes is still a good idea well in that case there is an easier way to recreate the AND. You can see from the original equation that Wi has an AND-like equation in the case that the parent nodes are both activating and inhibiting. Well one can simply include a negation of one of the parents and change the respective connection to inhibiting and the behavior of the system should be the same. Graphically this looks like this:

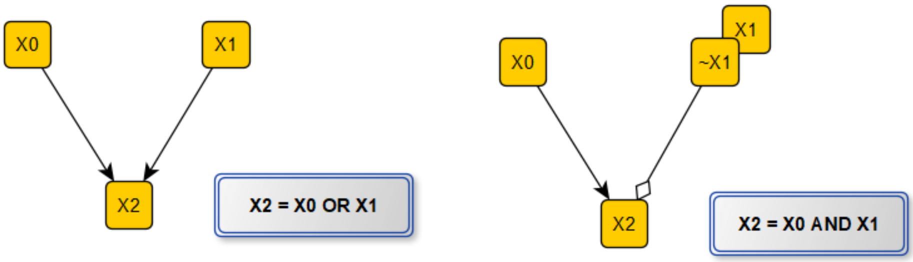

Where ~X1 means X1 negated. We remark that this is not true in general but it is true for the Mendoza and Xenarios [26] method. The truth table for the new configuration would be:

X0	X1	~X1	X2
0	0	1	0
0	1	0	0
1	0	1	0
1	1	0	1

The table corresponds to an AND between X0 and X1 but in a configuration such that the method can transform into a continuous system without any problem.

References

1. Robey, P. "Mesenchymal stem cells": fact or fiction, and implications in their therapeutic use. *F1000Research* **6** (2017).
2. Barrett, T. *et al.* NCBI GEO: archive for functional genomics data sets--update. *Nucleic acids research* **41,** D991-5 (2013).
3. Leung, V. Y. L. *et al.* SOX9 governs differentiation stage-specific gene expression in growth plate chondrocytes via direct concomitant transactivation and repression. *PLoS genetics* **7,** e1002356 (2011).
4. Lecka-Czernik, B. *et al.* Divergent effects of selective peroxisome proliferator-activated receptor-gamma 2 ligands on adipocyte versus osteoblast differentiation. *Endocrinology* **143,** 2376–2384 (2002).
5. MacArthur, B. D., Please, C. P. & Oreffo, R. O. C. Stochasticity and the molecular mechanisms of induced pluripotency. *PloS one* **3,** e3086 (2008).
6. Rosen, E. D. *et al.* C/EBPalpha induces adipogenesis through PPARgamma: a unified pathway. *Genes & development* **16,** 22–26 (2002).
7. Mackinlay, K. M. *et al.* An in vitro stem cell model of human epiblast and yolk sac interaction. *eLife* **10** (2021).
8. Tomasetti, C. *et al.* Role of stem-cell divisions in cancer risk. *Nature* **548,** E13-E14 (2017).
9. Karlstedt, K., Ahman, M. J., Anichtchik, O. V., Soinila, S. & Panula, P. Expression of the H3 receptor in the developing CNS and brown fat suggests novel roles for histamine. *Molecular and cellular neurosciences* **24,** 614–622 (2003).
10. Beyer, L. A. *et al.* Age-related changes in expression of CTL2/SLC44A2 and its isoforms in the mouse inner ear. *Hearing research* **282,** 63–68 (2011).
11. Calcinotto, A. *et al.* Cellular Senescence: Aging, Cancer, and Injury. *Physiological reviews* **99,** 1047–1078 (2019).
12. Li, M.-M. *et al.* Development of an oncogenic dedifferentiation SOX signature with prognostic significance in hepatocellular carcinoma. *BMC cancer* **19,** 851 (2019).
13. Leclerc, R. D. Survival of the sparsest: robust gene networks are parsimonious. *Molecular systems biology* **4,** 213 (2008).
14. Naseem, M., Kaltdorf, M., Hussain, A. & Dandekar, T. The impact of cytokinin on jasmonate-salicylate antagonism in Arabidopsis immunity against infection with *Pst* DC3000. *Plant signaling & behavior* **8,** doi: 10.4161/psb.26791 (2013).
15. Kaltdorf, M. & Naseem, M. How many salicylic acid receptors does a plant cell need? *Science signaling* **6,** jc3 (2013).

16. Naseem, M., Srivastava, M., Tehseen, M. & Ahmed, N. Auxin crosstalk to plant immune networks: a plant-pathogen interaction perspective. *Current protein & peptide science* **16,** 389–394 (2015).
17. Naseem, M. *et al.* Integrated systems view on networking by hormones in Arabidopsis immunity reveals multiple crosstalk for cytokinin. *The Plant cell* **24,** 1793–1814 (2012).
18. Jones, J. D. G. & Dangl, J. L. The plant immune system. *Nature* **444,** 323–329 (2006).
19. Osmanoglu, Ö., Shams, S., Dandekar, T. & Naseem, M. Modeling Immune Dynamics in Plants Using JIMENA-Package. *Methods in molecular biology (Clifton, N.J.)* **2328,** 183–189 (2021).
20. Czakai, K. *et al.* Influence of Platelet-rich Plasma on the immune response of human monocyte-derived dendritic cells and macrophages stimulated with Aspergillus fumigatus. *International journal of medical microbiology : IJMM* **307,** 95–107 (2017).
21. Jiang, Z., Yu, S., Lin, H. & Bi, R. Expression and function of cartilage-derived pluripotent cells in joint development and repair. *Stem cell research & therapy* **11,** 111 (2020).
22. Mercer, T. R. *et al.* DNase I-hypersensitive exons colocalize with promoters and distal regulatory elements. *Nature genetics* **45,** 852–859 (2013).
23. Osnato, A. *et al.* TGFβ signalling is required to maintain pluripotency of human naïve pluripotent stem cells. *eLife* **10** (2021).
24. Kunz, M., Dandekar, T. & Naseem, M. A Systems Biology Methodology Combining Transcriptome and Interactome Datasets to Assess the Implications of Cytokinin Signaling for Plant Immune Networks. *Methods in molecular biology (Clifton, N.J.)* **1569,** 165–173 (2017).
25. Karl, S. & Dandekar, T. Convergence behaviour and Control in Non-Linear Biological Networks. *Scientific reports* **5,** 9746 (2015).
26. Mendoza, L. & Xenarios, I. A method for the generation of standardized qualitative dynamical systems of regulatory networks. *Theoretical biology & medical modelling* **3,** 13 (2006).
27. Breitenbach, T., Liang, C., Beyersdorf, N. & Dandekar, T. Analyzing pharmacological intervention points: A method to calculate external stimuli to switch between steady states in regulatory networks. *PLoS computational biology* **15,** e1007075 (2019).
28. Kraaijevanger, J., Lenferink, H. & Spijker, M. N. Stepsize restrictions for stability in the numerical solution of ordinary and partial differential equations. *Journal of Computational and Applied Mathematics* **20,** 67–81 (1987).
